# Quantum Dot Research in Breast Cancer: Challenges and Prospects

**DOI:** 10.3390/ma17092152

**Published:** 2024-05-04

**Authors:** Hossein Omidian, Renae L. Wilson, Luigi X. Cubeddu

**Affiliations:** Barry and Judy Silverman College of Pharmacy, Nova Southeastern University, Fort Lauderdale, FL 33328, USA; rw1273@mynsu.nova.edu (R.L.W.); lcubeddu@nova.edu (L.X.C.)

**Keywords:** quantum dots, breast cancer, targeted therapy, nanomedicine, personalized medicine

## Abstract

The multifaceted role of quantum dots (QDs) in breast cancer research highlights significant advancements in diagnostics, targeted therapy, and drug delivery systems. This comprehensive review addresses the development of precise imaging techniques for early cancer detection and the use of QDs in enhancing the specificity of therapeutic delivery, particularly in challenging cases like triple-negative breast cancer (TNBC). The paper also discusses the critical understanding of QDs’ interactions with cancer cells, offering insights into their potential for inducing cytotoxic effects and facilitating gene therapy. Limitations such as biocompatibility, toxicity concerns, and the transition from laboratory to clinical practice are critically analyzed. Future directions emphasize safer, non-toxic QD development, improved targeting mechanisms, and the integration of QDs into personalized medicine, aiming to overcome the current challenges and enhance breast cancer management.

## 1. Introduction

The pursuit of quantum dot (QD) products in breast cancer research addresses collective gaps and challenges across diagnostic and therapeutic domains. One primary area of focus is the development of targeted imaging and therapeutics, aiming to enhance the precision and efficacy of cancer treatment modalities. Specific challenges include the need for the targeted imaging of the HER2 receptor [1], the application of peptide-based quantum dot nanomedicine for tailored therapeutic delivery [2], and the co-delivery of anti-cancer siRNAs alongside imaging in triple-negative breast cancer (TNBC), addressing the lack of targeted therapies in such aggressive cancer types [3]. Recent advancements in semiconductor QDs highlight their superior fluorescence, resistance to photo-bleaching, and tunable light emission, potentially offering more sensitive and specific detection methods compared to traditional bioimaging technologies. Nevertheless, issues such as cytotoxicity and nonspecific uptake continue to constrain their broader application [4].

Furthermore, the research seeks to improve drug delivery systems, exemplified by advancements in photothermal therapy [5,6] and the development of dual stimuli-responsive nanoparticles for HER2-positive breast cancer therapy [7]. In addition, the use of carbon dots (CDs) has been identified as promising for enhancing natural imaging techniques and the delivery of targeted therapies. Despite being in the early stages of research, CDs have shown potential in bioimaging, drug discovery, and photodynamic therapy, although their societal impact and safety are still under extensive evaluation [8].

Intracellular dynamics and molecular mechanisms represent another critical research avenue, with a focus on understanding how QDs interact with and affect cancer cells at the molecular level. Challenges include studying the intracellular uptake of QDs [9], investigating induced epigenetic and genotoxic changes [10], assessing apoptosis induction [11], and exploring the internalization and recycling of folate receptors [12]. QD-based nanotechnology is also proving instrumental in constructing biomedical imaging platforms to study cancer cell behavior and the tumor microenvironment, both in vivo and in vitro, providing crucial insights into carcinogenesis, invasion, and metastasis [13].

The synthesis of nanomaterials and their cytotoxic effects is another pivotal area of research. Scientists are exploring the synthesis of carbon and cadmium sulfide quantum dots to evaluate their antitumor activities and cytotoxic effects against breast cancer cells [14,15]. Additionally, the potential of photodynamic therapy using quantum dot conjugates is being investigated to enhance treatment efficacy against breast cancer cells [16,17].

Advanced diagnostics and biomarker research are also central to the utilization of quantum dots in breast cancer, focusing on the early detection of mutations, such as those in the BRCA1 gene [18], and the quantitative analysis of cancer-related proteins like IGF1R [19]. Moreover, the use of QDs in glycophenotype analysis [20,21] and the detection of biomarker co-localization [22,23] highlight the role of these nanomaterials in enhancing the specificity and sensitivity of cancer diagnostics.

Addressing the therapeutic development challenges is essential for advancing breast cancer treatment. The absence of effective targeted chemotherapy for TNBC [24] and the overarching need for advanced theranostic approaches [25] are significant motivators behind quantum dot research. Moreover, studies on quantum dot detoxification mechanisms [26] and gene delivery systems [27], as well as efforts to inhibit breast cancer metastasis [28], underline the broad spectrum of challenges that quantum dot technology aims to address in the field of breast cancer research.

## 2. Quantum Dot Products for Breast Cancer

Quantum dots (QDs) are central to the advancements in nanotechnology, effectively bridging the disciplines of quantum mechanics and materials science. These tiny semiconductors, which are typically 2 to 10 nanometers in size, possess the unique ability to manipulate the behavior of light and electrons, displaying features not present in bulk materials.

Research on cadmium-based quantum dots has been comprehensive, with cadmium selenide (CdSe) quantum dots being fundamental in understanding quantum confinement effects. This research ranges from examining simple structures [29,30] to exploring more complex forms like core/shell CdSe@ZnS [31], and even bioconjugated variants tailored for specific uses [32,33,34]. Cadmium sulfide (CdS) quantum dots are also recognized for their electronic properties and versatility, progressing from basic to intricate biologically conjugated forms [35,36,37].

Concerns over cadmium’s toxicity led to the development of cadmium-free quantum dots. Among these, indium-based quantum dots stand out for their near-infrared emissions, which are vital for biomedical imaging [38]. Additionally, graphene quantum dots (GQDs) exemplify the adaptability of carbon, being explored for their unique electronic properties and their potential for integration with various materials [39,40,41,42,43].

Recent innovations in quantum dot technology include the creation of functionalized quantum dots, such as zinc oxide quantum dots for antibacterial purposes [44,45], and MXene-derived quantum dots that open new technological possibilities [46].

Quantum dots are notable for their exceptional versatility, allowing them to be customized for a wide array of applications. Their conjugation with antibodies turns them into precise tools for imaging and targeting, shedding light on cellular processes [47,48,49]. Furthermore, quantum dots can be modified with molecules like PEG to enhance their compatibility and functionality, broadening their applications from bioimaging to therapeutic interventions [1,44,47,50,51]. Advances in delivery systems using quantum dots highlight their potential in nanomedicine, with modifications that enhance biocompatibility and efficacy [44,45,52,53,54,55]. In targeted and co-delivery systems, quantum dots play a crucial role in advanced therapy, merging with biomolecules for precise therapeutic delivery, marking significant progress in medical nanotechnology [2,3,12,18,19,21,22,23,56,57].

Quantum dots represent a dynamic and influential element in nanotechnology’s progress, merging deep quantum mechanical insights with practical applications, and driving significant scientific and technological advancements. Their capacity for tailored design and application-specific utility features their importance in ongoing research and development, paving the way for innovative solutions.

## 3. Diagnostic and Imaging Enhancements

The research explored the application of far-red and near-infrared fluorescent quantum dots (QDs) for imaging tumors in nude mice with HER2/neu-positive breast cancer xenografts. These quantum dots were either coated with polyethylene glycol, making them bioinert, or they were attached to anti-HER2/neu scFv antibodies, targeting the tumors specifically. Both types of quantum dots successfully enabled the visualization of the tumors, with the anti-HER2/neu-conjugated quantum dots exhibiting a more intense fluorescent signal. This indicates that both bioinert and targeted quantum dots are effective in tumor imaging, with the targeted approach yielding a stronger fluorescence, specifically in the tumor areas [47]. Another development was the creation of a quantum dot (QD)-based double-color imaging technique to analyze HER2 levels in breast cancer cells and type IV collagen in the tumor matrix. This dual-imaging approach enabled the simultaneous observation of HER2 expression and matrix alterations, providing direct insights into the correlation between HER2 levels and the invasive characteristics and blood vessel penetration of breast cancer, thus underlining the significant link between HER2 expression and the aggressiveness of breast cancer [58].

In another study, graphene quantum dots conjugated with pembrolizumab (GQDs-pembrolizumab) were developed for breast cancer imaging. Pembrolizumab is known for targeting PD-1 and PD-L1 immune checkpoints. Various characterization techniques, including transmission electron microscopy (TEM), atomic force microscopy (AFM), and spectroscopy, were employed. These nanoconjugates were then radiolabeled with technetium-99m (Tc-99m) for imaging and assessing distribution in mice with 4T1 tumors, revealing high activity at the tumor site. This demonstrates the potential of using radiopharmaceutical-based quantum dots conjugated with immune checkpoint monoclonal antibodies for the specific targeting and imaging of breast cancer [39].

Further research utilized quantum dots conjugated with an EGFR antibody for the in vivo imaging of metastasis in human breast cancer cells within mice. This particular study concentrated on the visualization of liver metastasis by labeling MDA-MB231 breast cancer cells with QDs-EGFR antibody. Through this method, the metastasis of the cancer cells in the liver was successfully imaged using confocal microscopy, highlighting the technique’s capability in detecting and visualizing the process of single cancer cell metastasis in vivo [48]. Another study introduced indium-based near-infrared emitting quantum dots (In-based QDs) as a safer alternative to cadmium-based QDs for sentinel lymph node imaging in breast cancer, attributed to their reduced toxicity. The use of CuInS_2_/ZnS quantum dots and their distribution within the body were examined through inductively coupled plasma mass spectroscopy (ICP-MS). The findings indicated that these indium-based quantum dots exhibit low toxicity and are effective for imaging sentinel lymph nodes in breast cancer, irrespective of the metastatic status of the disease [38]. Another aspect of research discussed the application of quantum dots in optimizing sentinel lymph node mapping in breast cancer diagnosis and treatment. The study emphasized the benefits of using near-infrared quantum dots for image-guided tumor resection, while also addressing the challenges related to the elimination of quantum dots and the potential for their accumulation in the body. This highlights the utility of quantum dots in enhancing sentinel lymph node mapping and tumor margin delineation, albeit with considerations for their long-term effects [59].

The use of onion-like quantum dot quantum well (QDQW) heteronanocrystals (CdSe/ZnS/CdSe/ZnS) implanted at tumor sites to improve the sensitivity of thermal imaging for early-stage breast cancer detection was investigated. The study employed a two-dimensional finite element method to simulate bioheat transfer and assess the impact of the QDQW heteronanocrystals on the temperature profile at the tumor site. The results demonstrated enhanced sensitivity in thermal imaging for detecting early-stage breast cancer, facilitated by the unique structure of the quantum dots [60]. Cadmium selenium quantum dots (QDs) with and without silver coating were utilized to modulate light transmission. This technology has been adapted for medical use, specifically to differentiate between healthy and cancerous blood cells, which is crucial for the early detection of breast cancer. This advancement in detection technology could potentially transform how early breast cancer is diagnosed, thereby significantly enhancing treatment success and patient recovery rates [61].

Investigating quantum dot-based immunofluorescent (QD-IHC) technology for imaging HER2 and ER in breast cancer tissue microarrays aimed to elucidate tumor heterogeneity. The findings revealed that QD-IHC provides a sharper and more sensitive portrayal of breast cancer heterogeneity compared to conventional immunohistochemistry techniques, emphasizing its significance in advancing the comprehension of breast cancer’s intricate nature [62]. Additionally, the use of quantum dot bioconjugates for the in situ molecular profiling of breast cancer biomarkers was discussed, illustrating the capacity of multicolor quantum dots for the multiplexed and quantitative detection of tumor biomarkers in both cells and tissues, thereby offering substantial contributions to the field of molecular pathology [32]. The preferential accumulation of CdSe quantum dots in the cytoplasm of breast cancer cells highlights their potential as fluorescent labels for tumor imaging and therapeutic applications. This preferential localization suggests a promising avenue for the use of quantum dots in both tumor imaging and therapy, offering a targeted approach to cancer treatment [29]. The development of cadmium-free, biocompatible zinc-copper-indium-sulfide (ZnCuInS_2_) quantum dots was highlighted, noting their long fluorescence lifetimes and utility as bioimaging probes. These probes are particularly effective in suppressing cell autofluorescence and improving the signal–background ratio in highly autofluorescent human breast cancer cells. This advancement suggests a significant potential for these quantum dots in providing safer diagnostic options with improved imaging capabilities [63].

Table 1 summarizes key quantum dot (QD) enhancements for breast cancer imaging. Each entry outlines specific QD properties and their applications, including fluorescence, conjugation with antibodies, multiplexed detection capabilities, and suitability for in vivo imaging. The outcomes highlight effective tumor visualization, the precise targeting of biomarkers, and potential clinical applications in breast cancer molecular pathology.

## 4. Quantum Dot-Based Biomarker Detection in Breast Cancer

**Figure 1:**
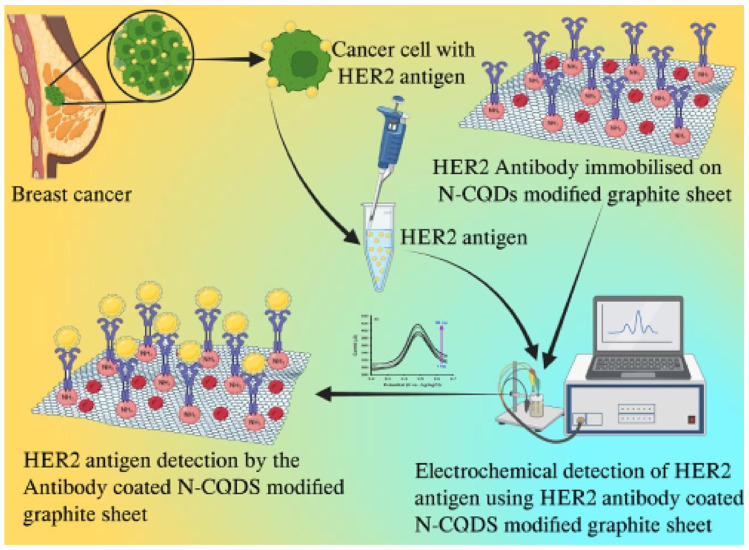
Shows a schematic illustration of nitrogen-enchanced carbon quantum dots (N-CQDs) on a coated graphite sheet (GS) substrate (N-CQDs/GS) for the electrochemical detection of breast cancer biomarker: human epidermal growth factor receptor 2 (HER2). This bovine serum albumin (BSA)-modified HER2 antibody/N-CQDs/GS immunoelectrode was highly stable and specific for untreated blood samples, and provided a linear response range and low detection limit of 0.1–1 ng/mL and 4.8 pg/mL, respectively [65].

Researchers developed nitrogen-enhanced carbon quantum dots (N-CQDs) on a graphite sheet substrate to create an immunoelectrode for the electrochemical detection of the HER2 breast cancer biomarker. The immunoelectrode, modified with bovine serum albumin (BSA) and a HER2 antibody, exhibited high sensitivity and specificity in detecting HER2 in blood samples, highlighting its potential for accurate breast cancer diagnosis (Figure 1) [65]. Quantum dot-based immunofluorescence technology was used for the quantitative analysis of HER2 expression in breast cancer. This approach involved creating a quantum dot HER2 probe kit and developing image acquisition and analysis software. When applied to clinical samples, this method provided the precise and sensitive detection of HER2, outperforming traditional diagnostic techniques [66]. Further research employed quantum dot-based nanotechnology and spectral analysis to quantify the total HER2 load in breast cancer tissues. This method combined molecular and macropathological indicators, such as HER2 levels and tumor size, to uncover the heterogeneity of breast cancer and identify new subtypes with varying prognoses. This advancement could lead to more personalized therapeutic approaches for patients with breast cancer [67].

The development of a disposable electrochemical immunosensor utilizing core/shell CdSe@ZnS quantum dots was aimed at detecting the extracellular domain of the human epidermal growth factor Receptor 2 (HER2-ECD) in human serum. Using bare screen-printed carbon electrodes as the transducer, this sensor demonstrated a low detection limit and reliable performance, making it a promising tool for screening HER2-ECD in serum samples [31]. Additionally, an assay using core/shell streptavidin-modified CdSe@ZnS quantum dots, combined with immunomagnetic beads, was developed for the voltammetric analysis of HER2-ECD and breast cancer cells in human serum. This method, which involved carboxylic acid-functionalized magnetic beads and a screen-printed carbon electrode, achieved the sensitive and selective detection of HER2-ECD and HER2-positive breast cancer cells, offering a robust approach for breast cancer diagnostics [33].

A lab-on-a-bead microarray design was introduced, utilizing quantum dots as fluorescent tags on beads for the quantitative detection of breast and ovarian cancer markers (CA 15-3, CEA, and CA 125) in human serum. Employing flow cytometry for marker identification, this system facilitated the multiplexed analysis of cancer markers in serum, providing clear differentiation between antigen levels and enhancing the accuracy of cancer diagnosis [68]. An innovative immunoassay was introduced that employs gold nanospears electrochemically assembled onto thiolated graphene quantum dots (CysA/GQDs) for detecting the breast cancer-specific carbohydrate antigen CA 15-3. This hybrid interface effectively immobilized the CA 15-3 antigens, enhancing the bioactivity and stability of the assay. Its efficacy in detecting CA 15-3 was validated in human plasma and MCF-7 cell lysates, with the assay’s construction and performance evaluated using various spectroscopic and electrochemical techniques. This method achieved the ultrasensitive detection of CA 15-3 with high specificity, proving its potential for early breast cancer diagnosis [69].

The development of a quantum dot (QD)-based microfluidic method for quantifying multiple biomarkers in breast cancer cells marked a significant advancement in diagnostic technologies. This method, known as quantum dot-based microfluidic multiple biomarker quantification (QD-MMBQ), enables the immunochemical labeling of over eight proteins on cell blocks in less than an hour. It offers the enhanced quantification of multiple biomarkers with reduced nonspecific binding compared to traditional methods, demonstrating its efficiency and effectiveness in cancer diagnostics [70].

Researchers also developed an electrochemical biosensor composed of a film of gold nanoparticles, graphene quantum dots, and graphene oxide (AuNPs/GQDs/GO). This biosensor, designed for the simultaneous detection of multiple clinically relevant microRNAs in breast cancer, utilizes anthraquinone (AQ), methylene blue (MB), and polydopamine (PDA) as redox indicators. These indicators anchor captured miRNA probes, providing the ultrasensitive and selective detection of multiple microRNAs in human serum, thus enhancing the diagnostic accuracy for breast cancer [42].

Another innovative approach led to the creation of a nano-biosensor using cadmium selenide (CdSe) quantum dots (QDs) for detecting the 185delAG mutation in the BRCA1 gene. By attaching a P2-NH2 strand covalently to QDs with carboxylic acid groups, the QD-P2 complex was formed, which interacted with complementary DNA (cDNA) and mutated DNA (mDNA), resulting in emission property changes. This method identified mutations in the BRCA1 gene with high sensitivity, offering a promising tool for genetic screening in breast cancer [30].

**Figure 2:**
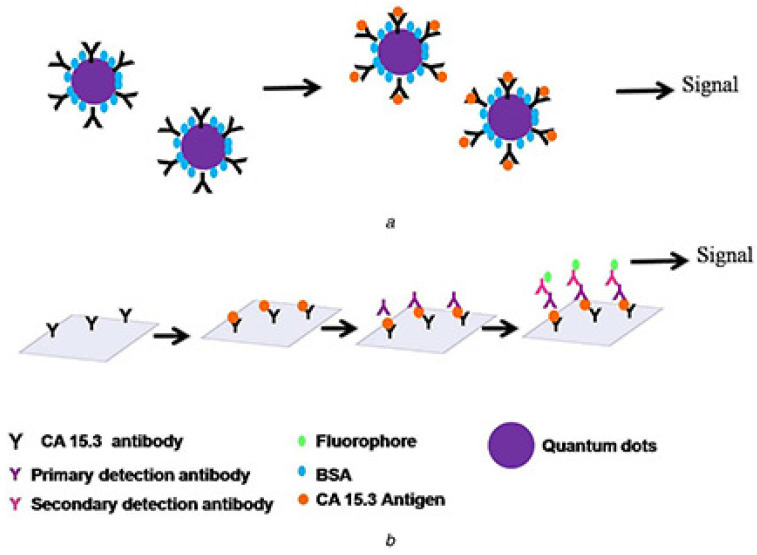
A schematic illustration of the comparison for the optical detection of CA 15.3 breast cancer antigen biomarkers. (**a**) Cds QD and (**b**) an automated kit. This Ab-Cys-CdS QD-based assay showed higher sensitivity and was able to detect the CA 15.3 biomarker even at a very low concentration of 0.002 KU/L when compared to the automated assays [35].

In addition, cadmium sulfide (CdS) quantum dots were utilized for the optical sensing of breast cancer antigen 15.3 (CA 15.3). By modifying the surface of CdS-QDs with cysteamine and tagging them with CA 15.3 antibodies, researchers were able to study antigen–antibody interactions using spectroscopic and microscopic techniques. This method demonstrated the ability to detect low concentrations of CA 15.3, showing potential for the development of sensitive biosensors for breast cancer detection (Figure 2) [35].

A bioconjugate of cadmium sulfide-selenide/zinc sulfide (CdSSe/ZnS) core/shell quantum dots with anti-estrogen alpha (ER alpha) antibody was developed for the molecular sensing of the breast cancer antigen ER alpha. Through optimized bioconjugation and fluorescence resonance energy transfer (FRET), this approach achieved high FRET efficiency and the sensitive detection of ER alpha, indicating a wide linear range for potential diagnostic applications [36].

Furthermore, the synthesis of thiol-stabilized water-soluble cadmium sulfide (CdS) quantum dots, functionalized with human transferrin protein siderophiline, was detailed. These customized quantum dots were tailored for visualizing MCF7 breast cancer cells, displaying intense green fluorescence upon specific excitation parameters. Through characterization via dynamic light scattering and fluorescence microscopy, these quantum dots facilitated the precise imaging of cancer cells with notable fluorescence while preserving satisfactory cell viability, underlining their promise for applications in targeted cancer diagnostics [37].

Researchers developed a novel electrochemiluminescence (ECL) sensor using an MXene-derived quantum dot (MQD) and gold nanobone (Au NB) heterostructure. Synthesized through a green process, this heterostructure enhanced the ECL signal for the detection of miRNA-26a in the serum of patients with triple-negative breast cancer. The sensor provided strong signal enhancement and efficient electron transfer, facilitating the sensitive detection of miRNA-26a, which is crucial for diagnosing this aggressive breast cancer subtype [46].

An N-doped graphene quantum dots (N-GQDs)-decorated tin sulfide (SnS_2_) nanocomposite was designed using a hydrothermal method for a non-enzymatic electrochemical sensor. This sensor aimed at the in situ monitoring of hydrogen peroxide (H_2_O_2_) secreted by human breast cancer cells, leveraging the conductive properties of N-GQDs and SnS_2_ nanosheets. The sensor exhibited the efficient and stable sensing of H_2_O_2_, with a wide detection range and low detection limit, offering a promising approach for monitoring cancer cell activity [41].

Quantitative spectral analysis was employed to molecularly classify breast cancer, utilizing quantum dots for the examination of hormone receptors (HRs) and human epidermal growth factor receptor 2 (HER2). Through the integration of quantitative data on HER2 and HRs, this investigation revealed the heterogeneity of breast cancer and delineated five molecular subtypes characterized by distinct prognoses. This effort established a novel molecular classification system for the disease [71].

A single quantum dot (QD)-based Forster resonance energy transfer (FRET) biosensor was developed for the sensitive detection of METTL3/14 complex activity in breast cancer tissues. This biosensor operates by preventing the cleavage of a substrate probe by the METTL3/14 complex, leading to nanostructure formation that facilitates FRET between the QD and a fluorophore. This approach allowed for the extremely sensitive detection of METTL3/14 complex activity, enabling detailed cell-level analysis and screening for potential inhibitors [72].

Table 2 presents quantum dot (QD)-based approaches for biomarker detection in breast cancer. These methods demonstrate enhanced sensitivity and specificity in detecting biomarkers such as HER2, microRNAs, and hormone receptors, enabling the accurate quantification and identification of breast cancer subtypes. The table highlights diverse applications, including electrochemical biosensing, fluorescent labeling, and spectral analysis, displaying the potential of QD-based technologies for precise and multiplexed biomarker detection in breast cancer research.

## 5. Quantum Dot Targeting and Detection of HER2 in Breast Cancer

**Figure 3:**
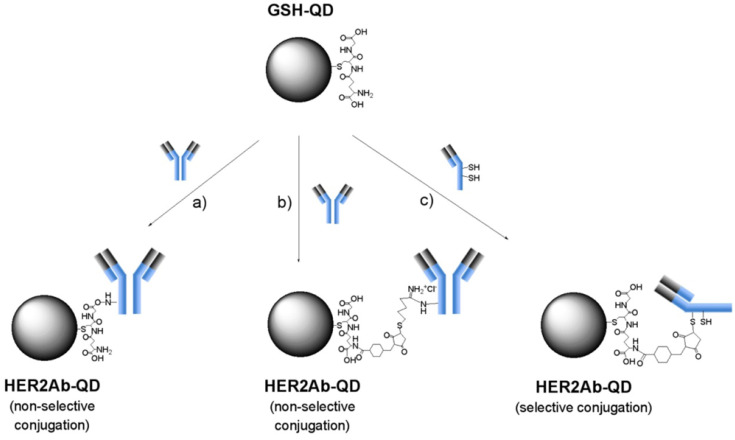
Shows a schematic illustration of the coupling reactions between GSH-QDs and anti-HER2 antibodies using coupling agents: (**a**) EDC/sulfo-NHS, (**b**) iminothiolane/sulfo-SMCC, and (**c**) sulfo-SMCC coupling. The HER2Ab-QD prepared with SMCC coupling was found to be the most effective probe for detecting HER2 expression in KPL-4 cells [34].

A new immunohistochemical technique was developed that employs quantum dot-conjugated trastuzumab for single-particle imaging to quantitatively assess the HER2 expression level in breast cancer tissues. This method allows for the precise calculation of quantum dot-conjugated trastuzumab particles binding specifically to cancer cells, thereby offering an accurate measurement of HER2 protein levels. This quantification correlates with the HER2 gene copy number and potential therapy outcomes, presenting a step forward in personalized cancer treatment [73]. Semiconductor quantum dots conjugated with monoclonal anti-HER2 antibody (Trastuzumab) were employed for the molecular imaging of breast cancer cells. These quantum dots were used to label the cell membranes of the HER2-overexpressing breast cancer cells, aiming to develop a method for in vivo fluorescent cancer imaging. This application showed a clear in vivo imaging of breast cancer cells, indicating a promising future for quantum dots in cancer imaging and drug tracking applications [74]. Furthermore, three types of anti-HER2 antibody-conjugated quantum dots (HER2Ab-QDs) were synthesized using different coupling agents. These conjugates utilized glutathione-coated CdSe/CdZnS quantum dots (GSH-QDs), characterized through various techniques such as dynamic light scattering and fluorescence correlation spectroscopy. The synthesized quantum dots proved to be effective probes for detecting HER2 expression in breast cancer cells, offering valuable tools for the fluorescence imaging of cancerous cells and potentially improving diagnostic and therapeutic strategies (Figure 3) [34].

**Figure 4:**
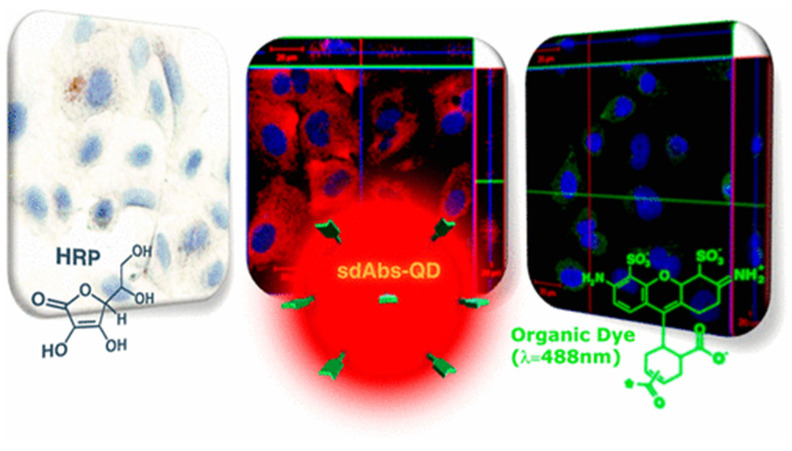
Showing an illustration of a highly sensitive ultra-small and bright nanoprobe based on QDs conjugated to a single-domain anti-HER2 (human epidermal growth factor receptor 2) antibody (sdAb) that achieved superior staining conjugate for the detection of lung cancer cell with differential HER2 expression. The sdAbs–QD conjugates provided superior staining showing potential for the development of a more sensitive assay for cancer biomarkers [73].

Another study presented a technique for assessing the labeling efficiency of the quantum dots used to label HER2 in breast cancer cells, utilizing correlative light and liquid-phase electron microscopy. This method enabled the correlation of fluorescence intensities with the molecular densities of the quantum dots, thereby refining the labeling process. The outcome was a notably high labeling efficiency, allowing for the precise quantification of the HER2 expression levels in cells, thereby improving the accuracy of cancer diagnosis and treatment planning [75]. Ultra-small quantum dots, conjugated to single-domain anti-HER2 antibodies, were utilized for the immunolabeling of breast and lung cancer cell lines. This research compared the staining capabilities of these conjugates with traditional organic dyes, revealing a superior staining and detection sensitivity for HER2 in cancer cell lines. This finding highlights the potential of these quantum dot conjugates in improving early cancer biomarker detection (Figure 4) [76].

A quantum dot (QD)-based approach was developed for hyper multicolor high-content single-cell imaging cytometry, enabling the simultaneous monitoring and quantitative estimation of breast cancer receptors such as EGFR1, HER2, ER, and PR. This method, utilizing different QD-antibody conjugates, allows for the quantitative classification of breast cancer subtypes by analyzing their molecular profiles. It facilitated a detailed classification of breast cancer subtypes and uncovered significant tumor heterogeneity, thereby enhancing the understanding of breast cancer biology, and aiding in tailored treatment strategies [77].

A quantum dot-based detection system was introduced for tracking HER2 in breast cancer cells and tissues employing QD525 and HER2-specific monoclonal antibodies. This study demonstrated the superior optical properties and sensitivity of quantum dots compared to FITC dye, exhibiting higher fluorescence intensity and photostability. This enhanced sensitivity for HER2 detection suggests that quantum dots could provide a more effective means for studying cancer cell dynamics and aiding in targeted cancer therapy [78].

Researchers also developed a technique for the selective collection and detection of MCF-7 breast cancer cells using aptamer-functionalized magnetic beads and quantum dots. In this method, the Mucin 1 protein (MUC1) aptamer was attached to magnetic beads, and CdTe quantum dots were coupled with the nucleolin aptamer AS1411 and coated on silica nanoparticles. This dual-aptamer bio-probe demonstrated an enhanced selectivity and sensitivity in detecting MCF-7 cells, achieving a detection limit of 85 cells per milliliter, showing promise for early cancer detection [79].

A single-cell analysis platform was fabricated using solid-state zinc-adsorbed carbon quantum dots (ZnCQDs) as an electrochemiluminescence (ECL) probe. This platform was designed for the detection and evaluation of CD44 expression on breast cancer cells, where ZnCQDs were combined with gold nanoparticles and magnetic beads to boost the ECL signal. Hyaluronic acid functionalization facilitated the specific targeting of cells, significantly enhancing the ECL intensity and enabling the precise evaluation of the CD44 expression levels, contributing to the advancement of cancer diagnostics [80].

Blue, fluorescent nitrogen-doped graphene quantum dots (N-GQDs) were synthesized from citric acid and diethylamine via a hydrothermal synthesis. These N-GQDs were conjugated with hyaluronic acid (HA) to target CD44 overexpressed on MCF-7 breast cancer cells. The process highlighted the importance of nitrogen doping for efficient amide bond formation, which facilitated HA conjugation and effective cell targeting. This method demonstrated a high fluorescence and low toxicity in cancer cell identification, suggesting its potential for accurate cancer diagnostics [43].

A water-soluble biomarker was developed using CuInS_2_/ZnS quantum dots (QDs) conjugated with an anti-Ki-67 monoclonal antibody. The initially hydrophobic QDs were coated with octadecyl amine and encapsulated with a biocompatible polymer before being conjugated to the antibodies. The resulting QD-Ki-67 probes enabled the detection of Ki-67 expression in breast cancer, offering new possibilities for bioimaging and diagnosis in the field of oncology [81].

Furthermore, different functionalized quantum dots were employed for dual-color in situ fluorescence imaging to study the coevolution of CD68 and CD47 in breast cancer. This research utilized high spatial resolution imaging to provide the observable evidence of the interactions between cancer cells and macrophages, enhancing the understanding of the tumor microenvironment and its implications for cancer progression and prognosis [82].

Table 3 highlights various quantum dot (QD) formulations and their applications in targeting and detecting HER2 in breast cancer. These QD formulations demonstrate an enhanced sensitivity and specificity in detecting HER2, facilitating the precise quantification of HER2 protein levels and enabling a clear in vivo imaging of breast cancer cells. The table highlights diverse approaches, including conjugation with specific antibodies, the optimization of labeling procedures, and the utilization of multicolor imaging techniques, underscoring the potential of QD-based technologies for targeted imaging and diagnosis in breast cancer research.

## 6. Quantum Dot Applications in Hormone and Protein Biomarker Detection

**Figure 5:**
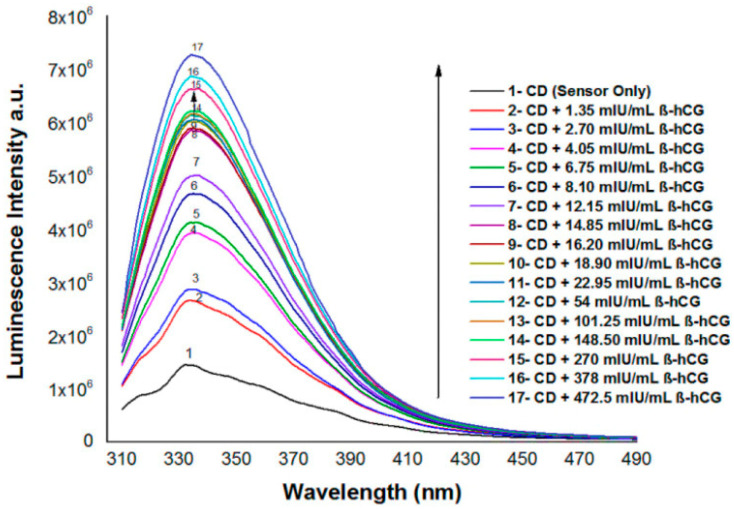
Shows results achieved for luminescence emission spectra of N/S-dopes CQDs in human chorionic gonadotropin β-hCG in the serum of the patients with breast and prostate cancer created from a low-cost highly selective method in the presence of different concentrations of β-hCG in DMSO at *λ*_ex_ = 288 nm. The method was successful in measuring the PCT in samples, used to assess biomarkers for cancer diseases in the body, achieving a dynamic range of 1.35–22.95 mU mL^−1^, with the limit of detection (LOD) and quantitation limit of detection (LOQ) of 0.235 and 0.670 mU mL^−1^, respectively [83].

A highly selective optical sensor based on nitrogen- and sulfur-doped carbon quantum dots (N/S-doped CQDs) was developed to assess the levels of human chorionic gonadotropin beta-hCG in the serum of the patients with breast and prostate cancer. The sensor, engineered to emit blue luminescence at specific wavelengths, responds to the varying concentrations of beta-hCG, offering a sensitive method for the assessment of this cancer biomarker in serum samples. This innovation promises the highly selective and sensitive detection of beta-hCG levels with low detection thresholds (Figure 5) [83].

An electrochemical biosensor was devised for the ultrasensitive detection of MCF-7 breast cancer cells in human serum employing nitrogen-doped graphene quantum dots (NGQDs) and phytohemagglutinin-L (PHA-L) deposited on screen-printed electrodes. Synthesized via a microwave-assisted hydrothermal method and coupled with PHA-L, the NGQDs not only enhanced electrical conductivity but also exhibited selective targeting of MCF-7 cells, demonstrating the biosensor’s potential for early and precise breast cancer diagnosis. This advancement highlights the biosensor’s significant sensitivity and specificity, indicating potential breakthroughs in early cancer detection with exceptionally low detection limits [84].

Research on the effects of the surface modification of quantum dots (QDs) using PEG and BSA explored their impact on the viability and migration of triple-negative breast cancer cells. These QDs were prepared via a hydrothermal method and chemically modified to assess their optical performance and biological effects. The study aimed to shed light on the potential clinical application of QDs in breast cancer detection, offering insights into the design of optical sensors with enhanced luminescence sensitivity for detecting cancer biomarkers [51].

Researchers also designed a biomarker using water-soluble AgInS_2_/ZnS quantum dots (QDs) conjugated to the P53 monoclonal antibody, aiming to diagnose breast cancer. The quantum dot-based biomarker was crafted to provide targeted recognition ability, low toxicity, and robust light stability, facilitating the detection of the P53 protein expression in breast cancer cells through confocal laser scanning microscopy. This method demonstrated the potential for the targeted and stable imaging of P53 in breast cancer cells, enhancing early diagnosis with minimal toxicity [85].

For early-stage breast cancer cell detection, quantum dots (QDs) conjugated with two kinds of anti-HER2/neu antibodies were developed—one for labeling and the other for imaging. The process involved SK-BR3 cells, the first antibody, and the QD-conjugated second antibody, generating fluorescent signals for microscopic analysis. This approach resulted in an enhanced imaging of breast cancer cells, offering brighter signals compared to those from organic dyes, and indicated a significant potential for improved diagnostic imaging [49].

Quantum dots were also applied for breast cancer cell detection, utilizing a trio of antibodies: anti-HER2/neu for capturing cells on silanized glass, anti-EpCAM for labeling, and a third antibody against anti-EpCAM conjugated to QDs for imaging. This method produced bright, fluorescent signals of captured SK-BR3 cells under a UV microscope, highlighting the enhanced potential of QDs in cell sensing and imaging for multiplexed immunological assays [86].

A study targeted protease-activated receptor 1 (PAR1) using anti-PAR1 antibody conjugated to quantum dots (QDs) for imaging and quantification in HER2-negative breast cancer tissues. The aim was to enhance the quantitative sensitivity of immunohistochemistry with QDs, correlating PAR1 expression with the likelihood of breast cancer recurrence post-surgery. This method enabled a more accurate monitoring of cancer progression and response to therapy, providing a predictive tool for assessing breast cancer recurrence [87].

Table 4 presents quantum dot (QD) applications in hormone and protein biomarker detection, particularly focusing on breast cancer research. These QD formulations demonstrate enhanced sensitivity and selectivity in detecting biomarkers such as beta-hCG, P53, HER2/neu, and EpCAM, enabling the accurate assessment and imaging of breast cancer cells. The table highlights diverse approaches, including conjugation with specific antibodies, surface modifications, and the utilization of fluorescent signal generators, underscoring the potential of QD-based technologies for precise biomarker detection in breast cancer diagnostics.

## 7. Quantum Dots for Targeted Drug Delivery in Breast Cancer

Researchers synthesized graphene quantum dots (GQDs) decorated with folic acid (FA) and methoxy polyethylene glycol (mPEG2000), which were then loaded with the drug tamoxifen (TMX). The characterization of this nano-drug revealed an average hydrodynamic diameter of 294.7 nm in water, with a dehydrated particle size ranging from 53 to 210 nm. This development showed that the tamoxifen-loaded GQDs were more toxic to cancer cells compared to tamoxifen alone, indicating a potential for enhanced cell monitoring and targeted therapy in breast cancer [88]. Graphene QDs conjugated with tamoxifen and folic acid were designed for targeted drug delivery, particularly in the treatment of breast cancer. These QDs aim to enhance the effectiveness of the drug tamoxifen by delivering it directly to cancer cells using the folic acid as a targeting agent. The pH-sensitive release mechanism of these QDs is particularly notable, as it ensures that the drug is released more significantly in the acidic environment of cancerous tissues compared to normal tissues, thus maximizing the therapeutic effects while minimizing side effects [89].

Glucosamine (GlcN)-conjugated graphene quantum dots (GQDs) loaded with curcumin (Cur) were fabricated for targeting breast cancer cells. These GQDs were synthesized from graphene oxide using an environmentally friendly oxidizing method, resulting in particles 20–30 nm in size with a pH-sensitive release behavior. This design facilitated the enhanced delivery and increased cytotoxicity of curcumin to cancer cells, offering precise targeting and tracking capabilities, which could improve the effectiveness of breast cancer treatments [90]. A graphene quantum dot (GQD)-based nanocarrier was developed, and labeled with Herceptin (HER) and beta-cyclodextrin (β-CD), to target and treat HER2-overexpressing breast cancer. The GQDs served as a part of a multifunctional drug delivery system, providing both therapeutic and diagnostic functions. This theranostic agent significantly enhanced anticancer activity through targeted delivery and controlled drug release in the acidic environment of cancer cells, illustrating a sophisticated approach to breast cancer treatment [53].

Highly luminescent carbon quantum dots (CQDs) were synthesized via the carbonization of citric acid and modified with transferrin (TF) to improve water solubility and facilitate the targeted delivery of doxorubicin (Dox) to breast cancer cells. The Dox-loaded TF-CQDs were characterized using various spectroscopic techniques, demonstrating an improved drug delivery efficiency and increased cytotoxicity to cancer cells, thus suggesting a promising avenue for enhancing the efficacy of breast cancer chemotherapy [91]. Graphene quantum dots (GQDs) were coated with a cationic polymer, poly(methacrylic acid-co-diallyldimethylammonium chloride) (PMA DDA), to investigate their potential in targeted drug delivery and the imaging of breast cancer. The chemical structure and particle size of these coated GQDs were analyzed using Fourier transform infrared spectroscopy (FT-IR) and Field Emission Scanning Electron Microscope (FE-SEM). The evaluations of in vitro toxicity and in vivo imaging in BALB/C mice with 4T1 breast cancer cells demonstrated that PMA DDA-coated GQDs could accumulate around cancer cells, highlighting their potential for effective breast cancer treatment and imaging [92].

The photodynamic therapy (PDT) capabilities of graphene quantum dots (GQDs) combined with methylene blue (MB) were explored, focusing on their potential to increase reactive oxygen species generation and eradicate breast cancer cells. The study evaluated the uptake and cytotoxic effects in MCF-7 breast cancer cells, discovering that methylene blue enhanced cytotoxicity and singlet oxygen generation in these cells, highlighting the potential of this combination to improve the efficacy of photodynamic therapy in treating breast cancer [93]. A photodynamic therapy agent was synthesized using 5,10,15-tris(5-bromo-2-thienyl),20(phenylcarboxy)porphyrin conjugated to graphene quantum dots, and its activity against MCF-7 breast cancer cells was studied. This conjugate proved suitable for photodynamic therapy, showing stable conjugation and significant anticancer activity, thereby offering a new avenue for effective breast cancer treatment through photodynamic therapy [94].

Researchers investigated the anticancer effects of silver graphene quantum dots (SQD) combined with 17-AAG and radiation on breast cancer cells. The study focused on assessing cell viability, apoptosis, and the expression levels of related genes and proteins. Findings indicated that this combination induced apoptosis and enhanced the effects of radiation, suggesting a promising strategy for combined cancer therapy, particularly in enhancing treatment efficacy [95]. Organotin (IV)-decorated nitrogen-doped graphene quantum dots (NGQDs) were developed for targeting and treating triple-negative breast cancer. These NGQDs, noted for their water solubility, chemical stability, and photoluminescence, demonstrated high cytotoxic potential and successful cellular uptake, marking them as potential candidates for targeted therapy and molecular imaging in the context of aggressive breast cancer types [96].

Doxorubicin was incorporated into liposomal nanoparticles containing quantum dots using reverse phase evaporation to form the liposome, which was then combined with graphite nanoparticles to create a nano-complex conjugated with doxorubicin. This nanoconjugated complex was found to significantly increase toxicity against breast cancer cells, thereby enhancing the drug’s efficacy and offering a promising approach to improve therapeutic outcomes [54]. Fluorescent carbon dots were engineered for bioimaging applications, with a focus on studying the interaction of anticancer drugs at the subcellular level. This technology helps visualize how drugs interact within cells, providing valuable insights that could aid in the design and development of new anticancer drugs. Such detailed imaging is crucial for researchers to understand the mechanisms of drug action, which could lead to more effective treatments with fewer side effects [97]. Dacarbazine was encapsulated within fucose-based carbon quantum dots, which were subsequently coated with exosomes derived from breast cancer cells. This strategy aimed to enhance the targeted delivery and therapeutic efficacy of the chemotherapeutic agent, leading to an improved antitumor targeting and efficacy with controlled drug release and cellular uptake, demonstrating a sophisticated approach to cancer treatment [98].

Zinc oxide quantum dots (ZnO QDs) were functionalized to prevent agglomeration in aqueous media and evaluated for their in vitro cytotoxic effects on breast and colon cancer cell lines. The surface functionalization utilized polymers, oily herbal fatty acids, polyethylene glycol (PEG), and organosilanes. These modified ZnO QDs showed low toxicity in normal cells while demonstrating the potential for use in cancer chemotherapy, suggesting they could be a viable option for targeting cancer cells with minimal side effects [44]. The innovative zinc oxide quantum dot nanoparticles were investigated for their anticancer properties against breast cancer stem-like cells, focusing on their impact on cell proliferation, apoptosis, and stemness markers. Synthesized and characterized ZnO nanofluids were assessed for their biological activities on mammospheres enriched with breast cancer stem-like cells. The results indicated that these nanoparticles could decrease stemness markers, induce apoptosis, and inhibit the JAK/STAT pathway, highlighting their potential as a novel therapeutic agent against breast cancer stem-like cells [45]. Graphene quantum dots functionalized with hyaluronic acid offer a targeted drug delivery system specifically aimed at breast cancer cells. By binding to receptors on cancer cells, these QDs can deliver drugs more efficiently and reduce toxicity, which is beneficial for improving patient outcomes. The targeted approach not only enhances the effectiveness of the treatment but also minimizes the impact on healthy cells, making treatments safer and potentially more effective [99].

Table 5 highlights the utilization of graphene quantum dots (GQDs) for targeted drug delivery in breast cancer treatment. These formulations demonstrate enhanced drug release efficiency, the specific targeting of cancer cells, and controlled drug release in the acidic tumor microenvironment. The table displays various strategies such as conjugation with targeting ligands, pH-sensitive release systems, and combination therapies, indicating the potential of GQDs in improving the efficacy and specificity of breast cancer treatment.

## 8. Theranostic Applications of Quantum Dots in Breast Cancer

Researchers developed tumor-targeted theranostic nanocapsules co-loaded with celecoxib and honokiol to enhance the treatment of breast cancer. These nanocapsules were constructed by assembling anionic CD44-targeting chondroitin sulfate and cationic lactoferrin onto the surface of a positively charged oily core. For imaging purposes, mercaptopropionic acid-capped cadmium telluride quantum dots were attached to the lactoferrin. This innovative approach resulted in nanocapsules that not only facilitated imaging but also exhibited increased cytotoxicity against cancer cells, demonstrating superior in vivo antitumor efficacy [100]. A novel theranostic hybrid peptide was synthesized for the simultaneous diagnosis and therapy of cancer cells. It comprised a biotinylation site, a HER2/neu-binding motif, and a dockerin domain. This peptide was conjugated with quantum dots-streptavidin and magnetic nanoparticles-streptavidin, enhancing the detection and destruction of the HER2/neu-positive breast cancer cells through fluorescence and hyperthermia. This dual-functionality approach provided a powerful tool for the targeted treatment and real-time monitoring of breast cancer [101].

**Figure 6:**
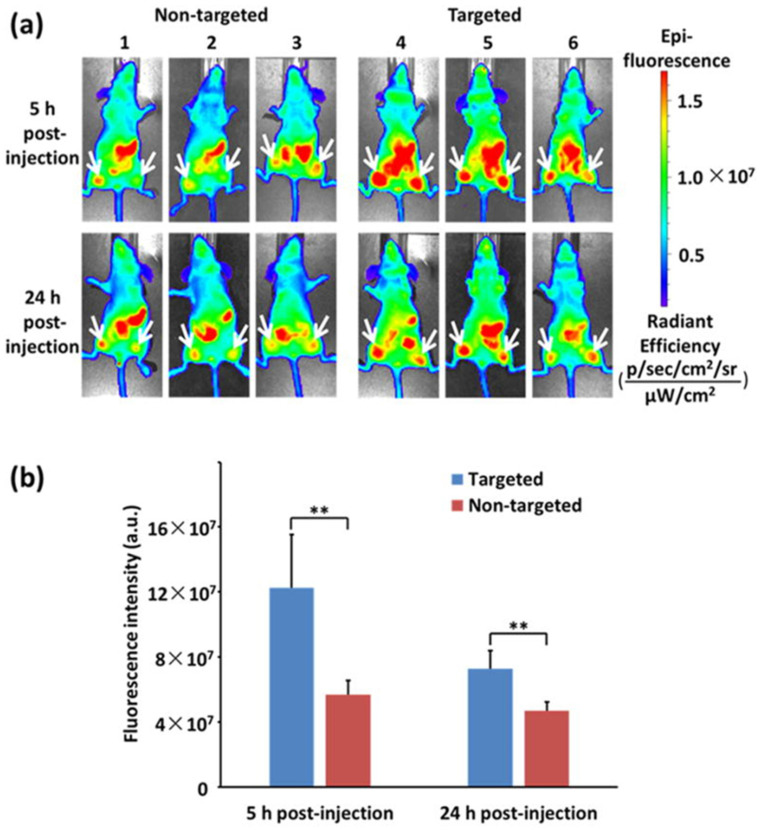
Shows images with results of targeted and non-targeted fluorescence with white arrows indicating the tumor sites. (**a**) Fluorescence images of MDA-MB-468 tumors in mice 1, 2, and 3 treated with non-targeted and targeted QD-PLA-PEG micelles in mice 4, 5, and 6. (**b**) Fluorescence intensity analysis of the QD-PLA-PEG micelles (targeted and non-targeted) at the MDA-MB-468 tumor sites (*n* = 6) in mice. ** Indicates *p* < 0.01.

In another advancement, quantum dot-based micelles were engineered, conjugated with an anti-epidermal growth factor receptor (EGFR) nanobody, and loaded with aminoflavone. The micelles utilized near-infrared fluorescence indium phosphate core/zinc sulfide shell quantum dots for in vivo imaging. The incorporation of the anti-EGFR nanobody significantly improved cellular uptake and cytotoxicity in EGFR-overexpressing triple-negative breast cancer cells, enhancing tumor targeting and treatment efficacy in an orthotopic xenograft model (Figure 6) [102]. 

Multilayered core/shell nanoprobes (MQQ-probe) were developed for the multimodality imaging of breast cancer, combining magnetic nanoparticles (MNPs) and quantum dots (QDs) within silica layers. These nanoprobes were conjugated with anti-HER2 antibodies to target cancer cells specifically and were employed for both in vitro and in vivo fluorescence and magnetic resonance imaging, enabling the comprehensive and targeted imaging of breast cancer tumors [103].

Quantum dot-based quantitative immunofluorescence was employed to detect and analyze the expression of the epidermal growth factor receptor (EGFR) in breast cancer tissue arrays. Utilizing quantum dots in immunohistochemistry allowed for a more precise quantitative analysis of EGFR, aiming to determine its prognostic value in breast cancer. This method helped identify the significance of EGFR expression in specific subgroups of the patients with breast cancer, enhancing the understanding of its role in cancer prognosis [104]. The expression of TOP2A in triple-negative breast cancer was quantitatively detected and analyzed using quantum dot-based immunofluorescent imaging. This approach, compared with conventional immunohistochemistry, validated the enhanced accuracy and prognostic significance of TOP2A expression, establishing it as an independent prognostic indicator in triple-negative breast cancer [105]. The in situ molecular imaging and quantitative analysis of EGFR and collagen IV were performed in triple-negative breast cancer using quantum dot-based technology. This research integrated essential tumor microenvironment components with cancer cell molecules to explore the invasion mechanism in triple-negative breast cancer and assess the prognostic value of the EGFR and collagen IV ratio. The findings revealed a negative correlation between EGFR and collagen IV, with the ratio offering significant prognostic insights for patient outcomes [106].

A double-color in situ quantitative imaging method based on quantum dots was developed to analyze the co-expression of Ki67 and HER2 in breast cancer. This technique utilized quantum dot-based fluorescent immunostaining, allowing for the simultaneous visualization of Ki67 (displayed as red fluorescence in the nucleus) and HER2 (depicted as green fluorescence on the cell membrane). The study quantitatively assessed the individual and combined impacts of these molecular expressions on breast cancer prognosis, offering insights into their roles in cancer development and potential treatment targets [107]. A multiple imaging technique using quantum dots was developed for the quantitative and in situ analysis of Ki67 and cytokeratin in breast cancer. By employing quantum dots to stain nuclear Ki67 and cytoplasmic cytokeratin, this method enabled the simultaneous quantitative analysis of these markers, improving the assessment of Ki67 in breast cancer diagnosis and prognosis [108].

Quantum dot nanoprobe-based high-content monitoring was employed to evaluate the effectiveness of capsaicin in inhibiting the growth of breast cancer stem cells. Using highly sensitive quantum dot–antibody nanoprobes, the study observed NICD translocation and apoptotic cell death at the single-cell level. This method demonstrated effective targeting and induced apoptosis in breast cancer stem cells, providing valuable data on the therapeutic potential of capsaicin against cancer stem cells [109]. Furthermore, quantum dots were utilized to image and detect the chemokine (C-C motif) ligand 5 (CCL5) and collagen IV in luminal B (HER2-negative) breast cancer, with the aim of calculating the CCL5/collagen IV ratio to determine its prognostic value. This quantitative imaging approach demonstrated that the CCL5/collagen IV ratio has a significant prognostic value for disease-free survival in patients with breast cancer, providing valuable information for tailoring patient-specific therapeutic strategies [110]. To effectively address the issue of poor targeting and increase specificity, a multifunctional nanoplatform called niosomes (NIO) was developed. This platform co-loaded paclitaxel (PTX) and quantum dots (QD) as bioimaging agents, along with hyaluronic acid (HA) (HN@QPS). The study demonstrated that this formulation led to an enhanced apoptosis rate of over 70% in MCF-7 breast cancer cells while showing no significant cytotoxicity on HHF-2 normal cells. This preliminary research highlights the potential of HN@QPS as an efficient targeted-dual drug delivery nanotheranostic agent against breast cancer [111].

Table 6 outlines the theranostic applications of quantum dots (QDs) in breast cancer management. These applications include targeted imaging, therapy, and the simultaneous detection of biomarkers, displaying the multifunctional capabilities of QDs in breast cancer research. The table demonstrates enhanced tumor targeting, treatment efficacy, and prognostic value through various QD-based approaches, emphasizing their potential for personalized medicine and improved patient outcomes in breast cancer care.

## 9. Targeted Theranostics and Imaging with Quantum Dots in Breast Cancer

Quantum dots (QDs) were conjugated with Herceptin^®®^, a fully human monoclonal antibody, to target the HER2 receptor in breast cancer therapy. These QDs, synthesized through various methods including top-down, bottom-up, and synthetic processes, were conjugated to Herceptin^®®^ to enable precise localization within cancer cells for bioimaging. This strategy facilitated the specific targeting and imaging of HER2 receptors, leading to the apoptosis of cancer cells, thus indicating a promising approach for treating HER2-positive breast cancer [1]. CdSe/ZnS core/shell quantum dots were linked with Herceptin to interact specifically with HER2-overexpressing breast cancer cells (SK-BR3), enhancing cell death through the binding of Herceptin to the HER-2 receptor on the cell membrane. This interaction was evaluated, showing that Herceptin-conjugated QDs specifically targeted and induced apoptosis in SK-BR3 breast cancer cells, while sparing non-cancerous cells (KB), highlighting their potential for selective cancer therapy [9].

Semiconductor quantum dots were attached to a specific peptide sequence (LTVSPWY) to achieve targeted delivery to human breast cancer cells. The conjugation exploited the unique chemical and detection characteristics of quantum dots, showing that peptide-conjugated QDs effectively targeted breast cancer cells, underscoring their potential for both diagnostic and therapeutic applications [2]. Carbon quantum dots (CDs) derived from sweet lemon peel were conjugated with polyamidoamine (PAMAM) dendrimers to create CD-PAMAM conjugates. These were further linked with the RGDS peptide to target ανβ3 integrin in TNBC cells, exhibiting efficient gene delivery and selective cancer cell targeting, suggesting a potential non-viral vector for diagnosis and gene therapy of TNBC [24].

Lipid nanocarriers guided by an anti-EGF receptor aptamer, encapsulating quantum dots and siRNAs, were developed for the diagnosis and treatment of triple-negative breast cancer (TNBC). These aptamer-coupled nanocarriers demonstrated an enhanced targeting and therapeutic efficacy, effectively delivering siRNAs and QDs to cancer cells, thus inhibiting tumor growth and metastasis while also facilitating tumor imaging [3]. Nitrogen-doped carbon quantum dots (N-CQDs) were prepared via a hydrothermal method and conjugated with quinic acid for the targeted delivery of the chemotherapy drug gemcitabine to breast cancer cells. The quinic acid-conjugated N-CQDs exhibited high tumor accumulation and outstanding luminescent properties, indicating their potential as effective theranostic agents [112]. Quantum dots were conjugated with calcitriol and MUC-1 antibodies to target inflammatory breast cancer (IBC) cells. A physiologically based pharmacokinetic model was developed to predict the behavior of these QDs in vivo, aiding in the optimization of quantum dot-based treatments for inflammatory breast cancer. This model provided valuable insights into tissue-specific QD concentrations in mice, supporting the development of targeted therapies for IBC [56]. Carbon QDs labeled with anti-PDL1 antibodies were used for targeted theranostic applications in the treatment of triple-negative breast cancer. These QDs enhance the effectiveness of the anti-PDL1 antibodies by delivering them directly to the tumor cells, thus promoting apoptosis and reducing tumor viability more effectively than the antibody treatment alone. This targeted approach is particularly important for treating the aggressive and difficult-to-treat forms of breast cancer [113].

Table 7 highlights targeted theranostic applications and imaging techniques using quantum dots (QDs) in breast cancer research. These applications cover the specific targeting of cancer cells, the enhanced delivery of therapeutic agents, and multimodal imaging capabilities, illustrating the potential of QDs for personalized medicine and improved cancer management. The table underscores the diverse strategies employed, including conjugation with targeting ligands, the utilization of lipid nanocarriers, and the exploration of photophysical characteristics for various therapeutic modalities, emphasizing the versatility and promise of QDs in breast cancer theranostic.

## 10. Quantum Dots for Metastasis Prediction and Inhibition in Breast Cancer

Silver sulfide quantum dots (Ag_2_S QDs) were conjugated with AMD3100 to target the CXCR4 receptor, a key player in breast cancer metastasis. This conjugation aimed to utilize the quantum dots for both imaging and inhibiting the spread of breast cancer, while also exploiting their photothermal properties to treat the primary tumor. The AMD3100-Ag_2_S QD probes were particularly effective in predicting and inhibiting metastasis, and their photothermal effect contributed significantly to reducing the size of the primary tumor, highlighting their potential as theranostic agents in the management of breast cancer [28].

Quantum dots were also conjugated with the anti-IGF1R antibody (AVE-1642) to detect and quantify IGF1R levels in breast cancer cells. These quantum dots were internalized by the cells through receptor-mediated endocytosis and localized within endosomes and the nucleus, facilitating not only the effective detection of IGF1R levels but also its downregulation. This process highlighted the potential of antibody-conjugated quantum dots as a traceable therapeutic approach, offering a dual benefit of detection and treatment in breast cancer management [19].

An advanced electrochemical DNA biosensor was developed using a double signal amplification strategy, employing cadmium telluride quantum dot-labeled DNA nanocomposites (3-QD@DNA NC) to hybridize with cleaved DNA probes for miRNA detection, specifically targeting the BRCA1 gene in human serum samples. This biosensor demonstrated high sensitivity for detecting BRCA1 gene mutations, offering a significant improvement in early breast cancer diagnostics and potentially allowing for earlier intervention and treatment [18].

Table 8 highlights the use of quantum dots (QDs) for metastasis prediction and inhibition in breast cancer. These applications include targeting specific molecular pathways, the sensitive detection of gene mutations, and facilitating receptor-mediated endocytosis for therapeutic intervention. The table emphasizes the potential of QDs in predicting and inhibiting breast cancer metastasis, enhancing early diagnostics, and offering traceable therapeutic approaches.

## 11. Cytotoxicity and Therapeutic Potential of Quantum Dots in Breast Cancer

Zinc oxide quantum dots (ZnO QDs) were synthesized to target breast cancer cells, specifically MCF-7 and MDA-MB-231, leveraging the acidic tumor microenvironment to enhance their therapeutic efficacy. These QDs promoted apoptosis and cell cycle arrest, showing significant cytotoxicity against the cancer cells even at low concentrations, indicating their potential as effective agents in targeted cancer therapy [114].

Graphene quantum dots (GQDs) were synthesized in three different forms (GQD, ortho-GQD, and meta-GQD) and assessed for their effects on breast cancer cell proliferation and apoptosis, particularly in estrogen receptor-positive cell lines. These GQDs were found to induce apoptosis and cell cycle arrest, highlighting their potential as selective agents for treating hormone-sensitive breast cancer subtypes [115].

Carbon quantum dots (CQDs) were synthesized using a specific chemical process and analyzed using X-ray diffraction (XRD) and high-resolution transmission electron microscopy (HRTEM). These CQDs demonstrated significant antitumor activity against breast cancer cell lines while exhibiting low toxicity to normal cells, presenting a promising avenue for cancer treatment that minimizes harm to healthy tissue [14].

Cadmium telluride quantum dots (QDs) were examined for their ability to cause epigenetic and genotoxic changes in human breast carcinoma cells. The QDs triggered global hypoacetylation and activated the p53 pathway, leading to cytotoxicity and potential epigenetic modifications. This study highlighted the profound impact of QD exposure on epigenetic regulation and stress response pathways in cancer cells, suggesting a complex interaction between nanomaterials and cellular processes [10]. The cytotoxic effects of various forms of cadmium telluride quantum dots (CdTe QDs), including high-yield CdTe QDs and CdTe/CdS core/shell QDs, were studied on human breast cancer cell lines. These QDs induced apoptosis in a dose-dependent manner, affirming the need for the careful evaluation of quantum dot dosages in therapeutic applications to maximize cancer cell targeting while minimizing potential side effects [11].

Cadmium sulfide (CdS) quantum dots were synthesized through a green process using waste tea leaves as a biosurfactant. These QDs showed strong fluorescence emission and cytotoxic effects on breast cancer cells, effectively inducing cell death, and arresting the cell cycle. This approach not only demonstrated the therapeutic potential of CdS QDs against breast cancer but also highlighted an innovative method of utilizing waste materials in the synthesis of effective cancer therapeutics [15].

Table 9 presents the cytotoxicity and therapeutic potential of quantum dots (QDs) in breast cancer treatment. These findings demonstrate the diverse cytotoxic effects of various QDs on breast cancer cells, including the induction of apoptosis, cell cycle arrest, and activation of stress response pathways. The table highlights the potential of QDs as promising therapeutic agents against breast cancer, highlighting their ability to induce cell death and inhibit cancer cell proliferation with varying degrees of toxicity.

## 12. Quantum Dot Applications in Histochemistry and Glycophenotype Analysis

Cadmium telluride (CdTe) quantum dots were conjugated with lectins, specifically Concanavalin A and Ulex europaeus agglutinin I, to assess the expression and distribution of glycoconjugates in various breast tissues. This innovative approach enabled the differentiation between normal and cancerous tissues based on their glycoconjugate profiles, offering a novel method for tissue characterization in breast cancer research [20]. Further study into the glycosylation patterns of breast tissues employed quantum dots conjugated with Cramoll lectin, which targets glucose/mannose residues. This investigation shed light on the changes in glycosylation between normal and cancerous breast tissues, revealing a marked increase in glucose/mannose residues within malignant tissues [21].

MPA-COOH-CdTe quantum dots, emitting at the wavelengths of 560 nm and 625 nm, were utilized to explore efflux mechanisms and toxicity in SK-BR-3 breast cancer cells, focusing on the role of ABC transporters in detoxification. The study found that ABC transporters influence the toxicity of quantum dots, with smaller-sized QDs prompting a greater expression of these transporters, suggesting size-dependent interactions that could inform the design of safer, more effective quantum dot-based therapies [26]. Folic acid (FA)-conjugated quantum dots were employed to investigate the internalization and recycling of folate receptors in breast cancer cells. Through fluorescence correlation spectroscopy (FCS) and saturation assays, the efficiency and specificity of these conjugates were evaluated, revealing the effective targeting and internalization of the FA-conjugated quantum dots by breast cancer cells. This variability in internalization and recycling across different cell types highlighted the delicate behavior of folate receptors in cancer cells, offering potential insights for targeted cancer therapies [12].

Anti-mortalin antibody-conjugated quantum dots were utilized to monitor the intercellular transfer between human mesenchymal stromal cells and breast cancer cells, emphasizing the necessity of direct cell-to-cell contact for this transfer. Observed in co-culture systems, this finding hinted at the potential mechanisms of intercellular communication and the role of mortalin in cancer progression [57]. In multiplex immunoprofiling, a combination of Alexa Fluor–quantum dot conjugates and chromogenic dyes was used to detect and localize breast cancer biomarkers within tissue sections. This approach facilitated the evaluation of co-expression and the spatial arrangement of prognostic markers, offering comprehensive insights into the tumor microenvironment. The elucidation of these biomarkers’ co-expression and spatial relationships provided valuable information for developing combinatorial therapeutic strategies in breast cancer treatment [22].

Table 10 highlights quantum dot (QD) applications in histochemistry and glycophenotype analysis in breast cancer research. These applications include distinguishing between normal and cancerous tissues based on glycoconjugate expression, analyzing glycophenotype, and studying cellular accumulation and interaction with transporters. Additionally, the table displays QDs’ effectiveness in targeting specific receptors, intercellular transfer between cells, and the multiplex immunoprofiling of biomarkers, offering insights into breast cancer pathology and therapeutic strategies.

## 13. Quantum Dot-Facilitated Cell Death Monitoring and Gene Delivery in Breast Cancer

**Figure 7:**
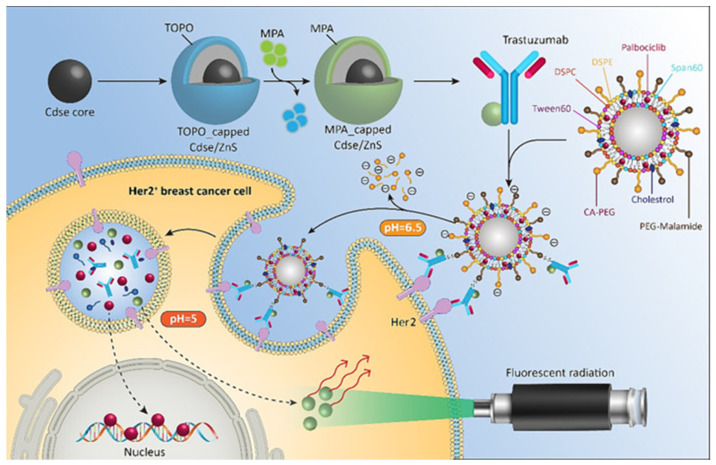
A schematic representation of the strategy, fabrication, and assessment methods used in this study to develop a targeted Pal-Trz-loaded smart niosome platform for treating HER2-positive BC cells [116].

Quantum dot-based total internal reflection fluorescence (TIRF) microscopy was utilized to monitor drug-induced cell death in breast cancer stem cells, focusing on the detection of phosphatidylserine and HMGB1 protein to differentiate between apoptotic and necrotic cell death. This technique allowed for the precise determination of drug concentrations effective against breast cancer stem cells, providing valuable insights into their susceptibility to treatment [23]. A formulation for a targeted and pH-sensitive niosomal system (pHSN) was developed, incorporating quantum dot (QD)-labeled Trastuzumab (Trz) molecules. This formulation aimed at the specific delivery of Palbociclib (Pal) via a two-phase pH-dependent release mechanism, resulting in an enhanced apoptosis induction in HER2-positive cells. The labeled Trz-conjugated Pal-pHSNs (Trz-Pal-pHSNs) exhibited significant cytotoxic effects, indicating their potential as personalized medicine for the treatment of HER2-positive breast cancer (Figure 7) [116].

Graphene oxide quantum dots (GOQD) were enhanced with polyethyleneimine (PEI) for gene delivery applications. The GOQD particles, covalently bonded with PEI, formed nanoparticles sized between 8 and 12 nm. This development was explored for its potential in gene therapy, specifically for delivering a suicide gene to breast cancer models, showing the successful gene delivery and induction of apoptosis in breast cancer cells, hence demonstrating its therapeutic potential [27].

Zn(II) phthalocyanines (ZnPcs) were linked with graphene quantum dots (GQDs) to investigate their photophysical properties and singlet oxygen generation capabilities for use in photodynamic therapy. The conjugation of ZnPcs with GQDs altered their photophysical properties, leading to an enhanced activity in photodynamic therapy against breast cancer cells [16]. Morpholino-substituted phthalocyanine (Pc) was conjugated to nitrogen and nitrogen–sulfur-doped graphene quantum dots for use in photo-sonodynamic therapy. The study focused on the reactive oxygen species (ROS) generation abilities of these conjugates under light and ultrasound exposure, revealing an improved ROS generation and therapeutic efficacy in combined therapy modalities for treating MCF-7 breast cancer cells [17].

PEGylated graphene quantum dots were conjugated with Herceptin to target HER2-positive breast cancer, with the nanoparticles designed to degrade in response to the glutathione and pH levels of the tumor microenvironment. This development demonstrated efficient tumor targeting, controlled drug release, and enhanced antitumor activity, offering a nuanced approach to breast cancer therapy [7].

Copper sulfide quantum dots (CuSQDs) were integrated into nano-graphene oxide (nGO) sheets to enhance photothermal therapy under near-infrared (NIR) laser exposure. This combination highlighted an enhanced cytotoxic effect against MCF-7 breast cancer cells, underlining the potential of photothermal therapy in effectively targeting and eliminating cancer cells [5]. Copper-doped carbon quantum dots (Cu-CDs) are emerging as a promising tool in the fight against breast cancer, showing remarkable capabilities in inhibiting the disease’s progression. Studies on MDA-MB-231 breast cancer cells reveal that Cu-CDs not only induce cell cycle arrest and promote apoptosis, but they also effectively reduce the migration and invasion of cancer cells. These findings underscore the potential of Cu-CDs to serve as the basis for new cancer treatment strategies that could effectively curb cancer progression with minimal adverse effects. Additionally, the anti-cancer properties of Cu-CDs are highlighted by their ability to mitigate oxidative stress mechanisms and curb other malignant behaviors, reinforcing their viability as a biocompatible and effective treatment option [117].

**Figure 8:**
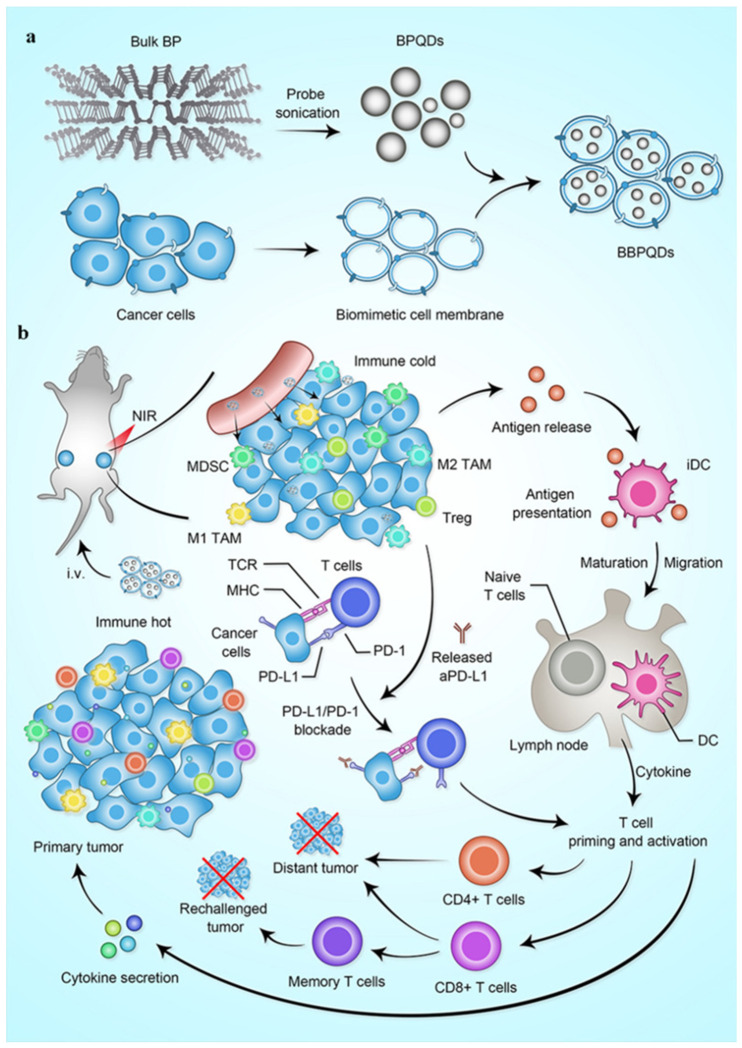
Shows: (**a**) An illustration of the synthesis process of BBPQDs. (**b**) The potential mechanism of BBPQDs-mediated PTT in combination with ICB therapy for anti-tumor treatment in cancer [6].

Biomimetic black phosphorus quantum dots (BBPQDs), coated with cancer cell membranes for targeted photothermal therapy and immunotherapy against triple-negative breast cancer, were studied for their photothermal efficiency and ability to activate immune responses. This approach effectively combined photothermal therapy with anti-PD-L1 immunotherapy, inhibiting tumor recurrence and metastasis, reprogramming the tumor microenvironment, and enhancing the antitumor immune response(Figure 8) [6]. 

Zinc oxide QDs as photosensitizers in photodynamic therapy use blue laser light to treat breast cancer cells. This combination therapy enhances the cytotoxic effects, promoting apoptosis and inhibiting cancer markers, thus offering a non-invasive treatment option that could improve patient outcomes through a synergistic approach [118].

Table 11 displays the role of quantum dots (QDs) in facilitating cell death monitoring and gene delivery in breast cancer. These applications include evaluating drug efficacies against breast cancer stem cells, inducing apoptosis through gene delivery, and enhancing therapeutic efficacy through photodynamic and photothermal therapies. The table emphasizes QDs’ potential in targeted therapy, controlled drug release, and the modulation of the tumor microenvironment, offering promising avenues for breast cancer treatment strategies.

## 14. Testing and Evaluation of the QD Products

To effectively leverage the capabilities of quantum dots (QDs) in scientific research, a series of comprehensive tests must be conducted. These tests span various disciplines and methodologies, each contributing to a deeper understanding of the properties, interactions, and potential applications of QDs.

Quantum Dot Synthesis and Characterization: This involves assessing optical, photophysical, and surface properties to optimize the QDs for specific applications [5,34,39,66,67,83,88,90,91,92,112,119]. Advanced characterization techniques such as transmission electron microscopy (TEM), Scanning Electron microscopy (SEM), Fouriertransform infrared spectroscopy (FTIR), X-ray diffraction (XRD), Ultraviolet-Visible spectroscopy (UV-Vis), photoluminescence (PL), high-resolution transmission electron microscopy (HRTEM), Field Emission Scanning Electron microscopy (FESEM), dynamic light scattering (DLS), photoluminescence spectroscopy (PL), and atomic force microscopy (AFM), are crucial for uncovering detailed structural and functional insights [5,14,15,30,34,35,79,88,89,90,112].

Molecular Imaging and Targeting: In molecular imaging and targeting, in vivo and in vitro studies are essential. These experiments utilize QD conjugation to explore imaging capabilities and biodistribution, aiming to understand how QDs travel and localize within biological systems [1,2,9,28,47,48,49,50,58,64,74,85,86,110]. Molecular imaging is further refined to quantify biomarker ratios, analyze statistical correlations, conduct survival analysis, and target specific receptors, enhancing the precision of diagnostics and targeted therapies [1,2,9,28,110].

Bioconjugation and Functionalization: For targeted therapy and imaging, the bioconjugation and functionalization of QDs are pivotal. Techniques to attach antibodies, peptides, and other targeting molecules to QDs enable researchers to tailor therapeutic agents that can precisely navigate to and interact with specific cellular targets [2,9,19,36,43,53,79,81,101,102].

Therapeutic Applications and Mechanisms: Exploring therapeutic applications involves evaluating drug delivery systems, therapeutic efficacy, and studying underlying mechanisms like apoptosis, and photothermal and photodynamic effects [1,3,6,7,10,16,17,25,96,115]. Mechanistic studies are critical as they delve into cellular responses, such as cell cycle progression and apoptosis, revealing how QDs influence biological processes at the molecular level [10,45,95,98,114,115].

Quantum Dots in Bioassays and Sensory Applications: QDs find extensive use in bioassays and sensory applications, where they are integrated into electrochemical, fluorescence, and enzyme-linked assays. These tests are designed to detect and quantify biological and chemical compounds, highlighting the adaptability and sensitivity of QDs in various assay contexts [18,27,33,36,46,63,65,72,84].

Cellular Uptake, Toxicity, and Interaction Studies: Understanding the interaction of QDs with biological systems necessitates studies on cellular uptake and toxicity. These investigations assess the biocompatibility and safety profile of QDs through the analyses of cellular uptake, cytotoxicity, and hemolytic activity [16,17,20,21,24,26,29]. Comprehensive toxicity assessments, both in vitro and in vivo, are fundamental to ascertain the potential adverse effects and ensure the safe application of QDs in biological environments [5,7,14,15,38,92,97].

Advanced Microscopy and Imaging Techniques: Advanced imaging techniques such as confocal microscopy, electron microscopy, and total internal reflection fluorescence (TIRF) microscopy play a crucial role in the detailed analysis of QDs at the cellular and molecular levels. These methods allow for the in-depth investigation of the interactions, localization, and functions of QDs within biological systems [7,9,12,22,23,32,48,49,57,74,75,85,102].

Spectroscopy and Photoluminescence Studies: Spectroscopic and photoluminescence studies are essential for characterizing the emission, absorption, and fluorescence properties of QDs. These analyses provide valuable information on the optical behavior of QDs, aiding in their development and application in diverse scientific fields [30,35,76,78,104,105,106,108].

## 15. Collective Outcomes

Quantum dots (QDs) have significantly advanced the field of breast cancer (BC) imaging and diagnosis. Their unique properties, such as small size, adjustable surface properties, and distinctive optical characteristics, have enabled non-invasive and precise imaging, facilitating the early and accurate diagnosis of breast cancer. Studies have shown that QDs enhance the capability for the in vivo imaging and specific fluorescence imaging of breast cancer cells, allowing the detailed tracking of tumor progression and therapy response [34,39,43,47,48,58,74,119]. Furthermore, QDs have been integrated with various materials to develop multimodal imaging techniques and theranostic applications, which improve the precision of cancer diagnosis and treatment. These advancements are particularly notable in the context of triple-negative breast cancer (TNBC), where combined therapy and diagnostics have shown potential to improve treatment outcomes [3,24,101,102].

QDs have demonstrated effective tumor targeting and provided an enhanced understanding of tumor heterogeneity and metastatic processes. Stronger tumor signals and high tumor uptake in various studies indicate the potential of QDs in enhancing BC diagnostics and understanding metastatic behaviors [32,39,47,48,58]. Additionally, QDs have facilitated the development of targeted cancer treatments, improving the delivery and efficacy of chemotherapeutic agents such as doxorubicin and tamoxifen. They have also been employed in novel therapeutic strategies, including photodynamic and photothermal therapies, demonstrating improved targeting and efficacy, and reducing potential side effects in breast cancer treatment [5,16,17,53,88,91,92,98,100].

The use of QDs in molecular profiling has significantly improved biomarker detection, aiding in the early and efficient detection of diseases and supporting the development of personalized therapy. Enhanced diagnostic accuracy for BC classification, prognosis prediction, and subtyping has enabled more personalized and effective treatment strategies. Advanced methods using QDs have facilitated the simultaneous detection of multiple biomarkers and genetic mutations, augmenting diagnostic efficiency and enabling early genetic screening for predisposition [22,29,30,31,33,40,42,46,62,65,66,67,68,69,70,71].

QDs conjugated with drugs or biomolecules have shown improved efficiency in targeted drug delivery and imaging in breast cancer treatment. These advancements have led to better therapeutic outcomes and have provided crucial insights into folate receptor dynamics and drug resistance mechanisms in breast cancer cells [12,26,56,112]. The development of QD-based biosensors has also demonstrated the ultrasensitive detection of breast cancer cells and genetic mutations, improving early diagnosis and potentially enhancing treatment outcomes [18,19,84].

QDs have induced cytotoxic effects and apoptosis in breast cancer cells, offering insights into their therapeutic potential and mechanisms of action. Research on the interaction between stromal and cancer cells facilitated by QDs has uncovered cellular communication mechanisms, suggesting new therapeutic targets. These findings have contributed to a better understanding of cancer cell behavior and the potential role of QDs in therapy design and optimization [10,11,57,114]. Additionally, QDs have been integrated into gene delivery systems, demonstrating their potential for targeted gene therapy in breast cancer, particularly in TNBC, and for monitoring drug-induced cell death in cancer stem cells. Investigations into glycocode changes in breast tissue using QDs have also suggested new methods for the early detection and monitoring of breast cancer progression [21,23,24,27].

## 16. Limitations

Concerns about the biocompatibility and long-term safety of quantum dots (QDs) are prominent, particularly regarding those containing heavy metals like cadmium. Research is essential to develop safer alternatives that are free from cadmium and other toxic materials, ensuring QDs can be safely used in clinical settings. The potential toxicity of these nanoparticles necessitates comprehensive studies to confirm their safety for biomedical applications. This need is stressed by the requirement for QDs that do not compromise patient health over time [10,11,30,35,63].

The journey from laboratory to clinic for QD-based technologies is fraught with challenges. Despite the promising results in preclinical studies, predominantly conducted on animal models, translating these findings to human applications is complex [7,32,64,85]. The process involves not only demonstrating the clinical efficacy and safety of QDs but also establishing scalable manufacturing processes for their widespread clinical use. Rigorous clinical trials and obtaining regulatory approval are critical steps in this journey, which is necessary to integrate QD-based technologies into standard cancer care protocols effectively.

The specificity and efficiency of QD-based targeting in cancer treatment are areas needing further optimization. Achieving consistent and reliable detection and treatment across different breast cancer types and stages remains a significant challenge. This task is compounded by the need to ensure that QD-based treatments selectively target cancer cells, minimizing impact on healthy cells. Furthermore, the intricate nature of cellular interactions in the tumor microenvironment adds another layer of complexity, influencing cancer progression and therapy resistance. These factors highlight the necessity for ongoing research to enhance the targeting accuracy and therapeutic efficacy of QD-based interventions.

The complexity and cost associated with the development of QD-based technologies may limit their accessibility and adoption in clinical practice. Sophisticated manufacturing processes and the potential excessive cost of these technologies could hinder their widespread clinical use. Additionally, the variability in tumor response to QD-based imaging and treatment necessitates further investigation into tumor heterogeneity and QD interactions, aiming to standardize these methods for broad clinical application. This endeavor is crucial for leveraging the full potential of QDs in improving breast cancer diagnosis, treatment, and patient outcomes.

## 17. Future Direction

Future directions in quantum dot (QD) research for breast cancer (BC) management emphasize the development of safer, non-toxic, and biocompatible QDs. This research aims to reduce potential toxicity and improve imaging efficiency for BC diagnosis, with a focus on developing cadmium-free QDs that mitigate safety concerns and facilitate clinical application [30,35,63]. Enhanced targeting and delivery systems are also crucial, with studies exploring the conjugation of QDs with various biomolecules to improve their targeting capabilities and therapeutic delivery, thereby enhancing imaging and treatment strategies [29,39,47].

Clinical trials and regulatory approval processes are pivotal for advancing QD technology. Comprehensive clinical trials are needed to establish the efficacy, safety, and regulatory approval of QD-based technologies, ensuring their integration into standard BC diagnosis and treatment protocols. The integration of QDs with personalized medicine is another critical area of focus. QDs hold the potential to facilitate personalized treatment plans through improved biomarker detection and molecular profiling, thereby aiding in the development of tailored and effective BC therapies [29,62,65,66].

The future of QD research also includes the creation of integrated platforms that combine QD-based imaging, biomarker detection, and genetic screening [1,9,32,35,65,112]. These platforms aim to offer comprehensive diagnostic and therapeutic solutions for BC, enhancing the precision and efficacy of treatments. Additionally, developing cost-effective production methods for QD-based technologies is essential to ensure their accessibility and adoption in routine clinical practice, particularly in resource-limited settings [15,23,36,39].

## 18. Conclusions

Quantum dots (QDs) have shown promising advancements in breast cancer imaging, diagnosis, and treatment, offering enhanced specificity, targeted therapy, and personalized medicine approaches. However, challenges such as biocompatibility, safety concerns, particularly with cadmium content, and the need for clinical validation remain significant. Future research should focus on developing non-toxic, biocompatible QDs, advancing targeted delivery systems, and conducting comprehensive clinical trials to ensure their efficacy and safety. The integration of QD-based technologies into theranostic platforms holds the potential for transforming breast cancer management, facilitating early detection, and enabling tailored treatment strategies, thereby improving patient outcomes in the face of breast cancer’s complexity and heterogeneity.

## Figures and Tables

**Table 1:** Quantum dot enhancements for breast cancer imaging.

QD Product and Its Properties	Outcome of the Study	Critical Assessment (Pros & Cons)	Ref
Cadmium selenide (CdSe) quantum dots	Preferential accumulation in the cytoplasm for tumor imaging/therapy	Pros: effective for imaging specific cellular components. Cons: potential toxicity due to cadmium content.	[29]
Multicolor quantum dot bioconjugates	Potential for in situ molecular profiling in molecular pathology	Pros: multifunctional with robust imaging capabilities. Cons: complexity in synthesis and potential bioaccumulation issues.	[32]
Indium-based near-infrared emitting quantum dots (CuInS_2_/ZnS)	Effective in SLN imaging; exhibited low toxicity	Pros: lower toxicity compared to other quantum dots. Cons: limited data on long-term effects and complete biocompatibility.	[38]
Graphene quantum dots conjugated with pembrolizumab	High tumor activity and specific targeting using a radiolabeled probe	Pros: the innovative combination of immunotherapy and imaging. Cons: complex synthesis, and unknown long-term biodistribution.	[39]
Cadmium selenide (CdSe) quantum dots conjugated with PEG and anti-HER2/neu scFv antibodies	The effective visualization of tumors with strong fluorescent signals	Pros: targeted imaging capabilities. Cons: the use of cadmium raises environmental and health concerns.	[47]
Quantum dots conjugated with EGFR antibody	Enabled the in vivo imaging of single cancer cell metastasis in the liver	Pros: high specificity and detailed imaging. Cons: potential issues with targeting efficiency and off-target effects.	[48]
Quantum dot-based double-color imaging system targeted at HER2	Linked HER2 expression to the invasive behavior of breast cancer cells	Pros: provides dual imaging options enhancing diagnostic capabilities. Cons: complexity in application and interpretation of results.	[58]
Near-infrared quantum dots	Potential for SLN mapping and tumor margin delineation, with slow elimination concerns	Pros: enhanced deep tissue imaging. Cons: risks associated with long-term body retention.	[59]
Onion-like quantum dot quantum well (QDQW) heteronanocrystal (CdSe/ZnS/CdSe/ZnS)	Enhanced thermal imaging sensitivity for early-stage breast cancer detection	Pros: improved imaging sensitivity. Cons: still in the early stages of research; long-term safety unknown.	[60]
Quantum dots conjugated with antibodies for HER2 and estrogen receptor (ER)	Provided a clearer and more sensitive depiction of breast cancer heterogeneity	Pros: increased clarity and sensitivity over conventional methods. Cons: requires precise control over antibody conjugation.	[62]
Cadmium-free, zinc copper indium sulfide (ZnCuInS_2_) quantum dots	Improved signal–background ratio in imaging, highlighting the potential for non-toxic diagnostics	Pros: non-toxic and biocompatible. Cons: may face challenges in commercial scale-up.	[63]
Quantum dots (CdSe, CdS) with a zinc sulfide (ZnS) shell, encapsulated in biocompatible coatings	The precise targeting of breast cancer masses and biomarkers	Pros: excellent specificity and biocompatibility. Cons: potential environmental impact and production costs.	[64]
Cadmium selenium QDs with and without silver coating, for light transmission modulation.	Differentiates between healthy and cancerous blood, enabling early breast cancer detection.	Pros: early detection capabilities enhance treatment outcomes. Cons: involves the complex synthesis and characterization of QDs; potential toxicity of cadmium.	[61]

**Table 2:** Quantum dot biomarker detection in breast cancer.

QD Product and Its Properties	Outcome of the Study	Critical Assessment (Pros & Cons)	Ref
Cadmium selenide (CdSe) quantum dots with carboxylic acid	Identified mutations in the BRCA1 gene with high sensitivity	Pros: high sensitivity and specificity for gene mutation detection. Cons: cadmium content raises toxicity concerns.	[30]
Core/shell CdSe@ZnS quantum dots with an electroactive label	Low detection limit and reliable HER2-ECD screening in serum	Pros: effective in detecting low concentrations of markers. Cons: limited to specific electrochemical detection setups.	[31]
Core/shell streptavidin-modified CdSe@ZnS quantum dots	The sensitive detection and selective identification of HER2-positive cells	Pros: high specificity for targeted cancer cell detection. Cons: potential streptavidin bioactivity issues.	[33]
Cadmium sulfide (CdS) quantum dots modified with cysteamine	Capable of detecting low concentrations of CA 15.3 for biosensor development	Pros: promising for early cancer marker detection. Cons: the stability and biocompatibility of CdS need clarification.	[35]
Cadmium sulfoselenide/zinc sulfide (CdSSe/ZnS) quantum dots	High FRET efficiency and the sensitive detection of ER alpha	Pros: effective for molecular sensing with a wide detection range. Cons: complex synthesis may limit practical use.	[36]
Cadmium sulfide quantum dots modified with human transferrin protein	Enabled the targeted imaging of cancer cells	Pros: specific targeting capabilities. Cons: concerns about cadmium toxicity remain.	[37]
Graphene quantum dots produced by electrochemical exfoliation	High sensitivity and low detection limit for CD44 detection	Pros: excellent sensitivity and broad application potential in biosensing. Cons: scale-up and reproducibility may be challenging.	[40]
N-doped graphene quantum dot-incorporated SnS_2_ nanosheets	The efficient and stable sensing of H_2_O_2_ with a wide detection range	Pros: robust and stable electrocatalytic performance. Cons: application beyond lab settings not proven.	[41]
Gold nanoparticles/graphene quantum dots/graphene oxide film composite	The ultrasensitive and selective detection of multiple microRNAs in serum	Pros: high sensitivity and selectivity in complex biological samples. Cons: complexity in fabrication and potential cost issues.	[42]
MXene-derived quantum dots @gold nanobone heterostructure	Strong signal enhancement for sensitive miRNA-26a detection	Pros: enhanced electrochemiluminescence for sensitive detection. Cons: integration into clinical settings needs further study.	[46]
Nitrogen-enhanced carbon quantum dots on graphite sheet	Excellent sensitivity and specificity for HER2 detection in blood	Pros: high performance with stability in sensing. Cons: limited information on the long-term viability of sensors.	[65]
Quantum dot-based probes for HER2 using immunofluorescent technology	Accurate and sensitive HER2 detection, superior to conventional methods	Pros: provides clear advantages over older diagnostic methods. Cons: dependence on specific instrumentation for analysis.	[66]
Quantum dots integrated with spectral analysis for HER2 load	Identified new BC subtypes enhancing personalized therapy	Pros: innovative approach to BC subtype classification. Cons: requires detailed spectral analysis capabilities.	[67]
Quantum dots as bead-bound fluorescent tags in flow cytometry	Enabled the multiplexed serum analysis of cancer markers	Pros: enhances multiplexing capabilities in flow cytometry. Cons: potential for overlap in fluorescence spectra.	[68]
Thiolated graphene quantum dots and gold nanospears	The ultrasensitive detection of CA 15-3 in plasma and cell lysates	Pros: high sensitivity and specificity in immunoassays. Cons: complex manufacturing process may impede widespread use.	[69]
Quantum dot-based labels in a microfluidic platform	Enhanced the quantification of biomarkers with less nonspecific binding	Pros: improves accuracy and reduces errors in microfluidic assays. Cons: microfluidic device integration may be technically demanding.	[70]
Quantum dots for quantitative spectral analysis in breast cancer classification	Established a new molecular classification revealing BC heterogeneity	Pros: leads to better-targeted therapies based on molecular profile. Cons: high-tech approach that may not be accessible everywhere.	[71]
Single quantum dot-based FRET biosensor for METTL3/14 complex	Extremely sensitive detection enabling cell-level analysis and screening	Pros: high sensitivity suitable for drug screening. Cons: FRET efficiency can vary depending on experimental conditions.	[72]

**Table 3:** Quantum dot targeting of HER2 in breast cancer.

QD Product and Its Properties	Outcome of the Study	Critical Assessment (Pros & Cons)	Ref
Cadmium selenide/cadmium zinc sulfide (CdSe/CdZnS) quantum dots conjugated with anti-HER2 antibody	Effective probes for detecting HER2 expression in breast cancer cells	Pros: the specific targeting of cancer markers. Cons: the potential toxicity of cadmium-based materials.	[34]
Nitrogen-doped graphene quantum dots (N-GQDs) conjugated with hyaluronic acid (HA)	High fluorescence and low toxicity in cancer cell identification	Pros: low toxicity and high fluorescence for safe diagnostics. Cons: the durability of fluorescence in clinical settings needs validation.	[43]
CdSe/ZnS quantum dots multiplexed with PEG and EGF	Quantified EGFR expression and monitored receptor regulation post-therapy	Pros: effective in the dynamic monitoring of therapy effects. Cons: complexity in clinical adaptation.	[50]
Quantum dot-conjugated trastuzumab	The precise quantification of HER2 protein levels, correlating with gene copy number and therapy outcomes	Pros: high precision in protein-level quantification. Cons: specificity to HER2 might limit broader application.	[73]
Polyethylene glycol coated quantum dot conjugated with anti-HER2 antibody (Trastuzumab)	A clear in vivo imaging of breast cancer cells	Pros: improved imaging quality and photostability. Cons: PEGylation might affect binding efficiency in some cases.	[74]
Quantum dots with high labeling efficiency (83%) for HER2	High labeling efficiency enabling the quantification of HER2 expression levels in cells	Pros: high efficiency in target labeling. Cons: limited information on the impact on cell viability.	[75]
Quantum dots conjugated with single-domain anti-HER2 antibodies	Superior staining and detection sensitivity for HER2 in cancer cell lines	Pros: enhanced detection sensitivity. Cons: the potential for nonspecific binding needs to be evaluated.	[76]
Quantum dot–antibody conjugates for EGFR1, HER2, ER, and PR	Facilitated the quantitative classification of BC subtypes and revealed significant tumor heterogeneity	Pros: allows for detailed single-cell analysis and subtype differentiation. Cons: complexity in simultaneous detection and analysis.	[77]
Quantum dot (QD525) conjugated with home-made HER2-specific monoclonal antibodies	Higher fluorescence intensity and stability, enhancing sensitivity for HER2 detection	Pros: improved optical properties for better diagnostic performance. Cons: consistency in antibody performance needs thorough assessment.	[78]
Cadmium telluride (CdTe) quantum dots on silica nanoparticles (SiO_2_ NPs)	Enhanced selectivity and sensitivity in detecting MCF-7 cells with a low detection limit	Pros: high selectivity and enhanced detection capabilities. Cons: cadmium toxicity remains a significant concern.	[79]
Solid-state zinc-adsorbed carbon quantum dots (ZnCQDs) on gold nanoparticles and magnetic beads	Significant enhancements in ECL intensity, evaluating CD44 expression levels	Pros: improved signal strength for sensitive analysis. Cons: the integration of multiple materials may affect reproducibility.	[80]
CuInS_2_/ZnS quantum dots conjugated with anti-Ki-67 monoclonal antibodies	The detection of Ki-67 expression in breast cancer, suitable for bioimaging and diagnosis	Pros: low toxicity with high quantum yield, suitable for long-term studies. Cons: specificity and long-term effects in in vivo applications need further research.	[81]
Dual-color functionalized quantum dots for the in situ fluorescence imaging of CD68 and CD47	Direct observable evidence of the coevolution of CD68 and CD47 in breast cancer, aiding in prognosis evaluation	Pros: high spatial resolution enhances the understanding of the tumor microenvironment. Cons: requires sophisticated imaging technology for analysis.	[82]

**Table 4:** Quantum dot applications in hormone and protein biomarker detection.

QD Product and Its Properties	Outcome of the Study	Critical Assessment (Pros & Cons)	Ref
Cadmium-based quantum dots conjugated with anti-HER2/neu antibodies	The enhanced imaging of breast cancer cells with brighter signals compared to organic dyes	Pros: higher brightness and stability than traditional dyes. Cons: toxicity concerns associated with cadmium.	[49]
N/S-doped CQDs modified with PEG and BSA, blue-shift in fluorescence	The accurate and highly selective assessment of beta-hCG levels in serum with enhanced luminescence sensitivity	Pros: improved selectivity and sensitivity for cancer marker detection. Cons: the long-term biocompatibility of modified CQDs needs further study.	[51]
N/S-doped carbon quantum dots (CQDs)	The highly selective and sensitive assessment of beta-hCG levels in serum with low detection limits	Pros: excellent sensitivity and specificity for detecting serum markers. Cons: the scalability and consistent production of doped CQDs may be challenging.	[83]
Nitrogen-doped graphene quantum dots (NGQDs) fabricated with lectin	Detected breast cancer cells in serum with high sensitivity and selectivity, extremely low detection limits	Pros: high performance in detecting cancer cells in complex biological fluids. Cons: specificity to breast cancer cells may limit broader diagnostic use.	[84]
AgInS2/ZnS quantum dots conjugated with P53 monoclonal antibody	The targeted and stable imaging of P53 in breast cancer cells with low toxicity	Pros: effective targeting and imaging with reduced toxicity. Cons: P53 targeting may not be applicable to all cancer types.	[85]
Semiconductor quantum dots conjugated with anti-HER2/neu and anti-EpCAM antibodies	The bright and specific imaging of breast cancer cells, potential for multiplexed assays	Pros: enhanced imaging capability, suitable for complex diagnostic applications. Cons: complexity in the conjugation process could affect reproducibility.	[86]
Streptavidin CdSe/ZnS quantum dots with PEG, EGF, and technetium-99m hydrazinonicotinamide	The quantitative monitoring of EGFR expression and response to cetuximab therapy in breast cancer	Pros: provides detailed quantitative data on therapy response. Cons: the use of radioactive elements like technetium-99m requires stringent handling and disposal measures.	[87]

**Table 5:** Graphene quantum dots for targeted drug delivery in breast cancer.

QD Product and Its Properties	Outcome of the Study	Critical Assessment (Pros & Cons)	Ref
Zinc oxide QDs modified with polymers and fatty acids	Low toxicity in normal cells with potential for cancer chemotherapy	Pros: specific cytotoxicity to cancer cells while sparing normal cells. Cons: potential issues with bioaccumulation and long-term effects.	[44]
Zinc oxide quantum dots against breast cancer stem-like cells	Decreased stemness markers, induced apoptosis, and inhibited JAK/STAT pathway	Pros: effective in targeting cancer stem cells. Cons: requires further validation in vivo to confirm efficacy and safety.	[45]
Quantum dots curcumin-loaded Eudragit RS 100 nanoparticles	The profound inhibition of colon and breast cancer cell growth	Pros: the effective delivery and enhanced action of curcumin. Cons: complexity in nanoparticle formulation may impact scalability.	[52]
GQD labeled with Herceptin and β-cyclodextrin for doxorubicin delivery	Enhanced anticancer activity through targeted delivery in the acidic cancer environment	Pros: targeted delivery enhances drug efficacy. Cons: dependency on the acidic environment for drug release could limit broader application.	[53]
Doxorubicin-loaded liposomal nanoparticles containing QDs	Increased toxicity significantly, improving drug efficacy	Pros: high drug loading efficiency and controlled release. Cons: increased complexity and potential for unintended toxicity.	[54]
Chitosan/carbon QDs/Fe_2_O_3_ nanocomposite containing curcumin	Effective targeted delivery and controlled drug release with cytotoxicity against MCF-7 cells	Pros: enhanced biocompatibility and stability. Cons: challenges in the clinical translation of composite materials.	[55]
Graphene quantum dots decorated with folic acid and mPEG2000, loaded with tamoxifen	Higher toxicity to cancer cells than free tamoxifen	Pros: enhances the effectiveness of tamoxifen and allows for cell monitoring. Cons: potential for folic acid to interfere with normal cellular functions.	[88]
Glucosamine-conjugated GQDs loaded with curcumin	Enhanced the delivery and cytotoxicity of curcumin to cancer cells with specific targeting	Pros: specific targeting and enhanced drug delivery. Cons: the confirmation of long-term safety and effectiveness needed.	[90]
CQDs modified by transferrin for doxorubicin delivery	Improved drug delivery efficiency and increased cytotoxicity in cancer cells	Pros: enhanced interaction with cancer cell receptors. Cons: potential for immune reaction or side effects due to transferrin modification.	[91]
GQDs coated with cationic polymer PMA DDA, imprinted with doxorubicin	Targeted drug delivery and imaging capabilities in vivo	Pros: effective targeting and dual functionality for therapy and diagnosis. Cons: complexity in synthesis could affect reproducibility and scalability.	[92]
GQDs combined with methylene blue for photodynamic therapy (PDT)	Superior cytotoxicity and singlet oxygen generation in breast cancer cells	Pros: the effective generation of reactive oxygen species for cancer treatment. Cons: specific conditions needed for optimal PDT performance.	[93]
Porphyrin-conjugated GQDs for photodynamic therapy (PDT)	Suitable for PDT with stable conjugates and significant anticancer activity	Pros: high stability and effective in generating singlet oxygen. Cons: specificity and delivery to tumor sites may need further optimization.	[94]
Silver graphene quantum dot (SQD) combined with 17-AAG and radiation	Induced apoptosis and increased radiation effects, potential for combined therapy	Pros: enhances the effectiveness of conventional therapies. Cons: potential for increased side effects with combined therapies.	[95]
Organotin(IV)-decorated NGQDs for the delivery of organotin(IV) compounds	High cytotoxic potential and successful cellular uptake for targeted cancer therapy	Pros: effective delivery and significant cytotoxic effects. Cons: the toxicity profile and biodegradability of organotin compounds need a thorough evaluation.	[96]
Dacarbazine-primed carbon QDs coated with breast cancer cell-derived exosomes	Enhanced tumor targeting and efficacy with controlled drug release	Pros: innovative approach enhancing specificity and efficacy. Cons: complex manufacturing process and the potential immunogenicity of exosomes.	[98]
Graphene QDs conjugated with tamoxifen and folic acid, used for targeted drug delivery.	Enhanced the killing of breast cancer cells; pH-sensitive release showing significant release differences between cancerous and normal pH environments.	Pros: targeted delivery reduces systemic side effects; enhanced drug efficacy. Cons: complex conjugation process; long-term effects and in vivo behavior remain to be fully evaluated.	[89]
Fluorescent carbon dots designed for bioimaging to study the action of anticancer drugs within cells.	Visualizes drug interactions at the subcellular level, revealing the mechanisms of anticancer drugs, potentially aiding drug design.	Pros: non-toxic and suitable for detailed bioimaging. Cons: still in the developmental stage; clinical applications and scalability need further study.	[97]

**Table 6:** Theranostic applications of quantum dots in breast cancer.

QD Product and Its Properties	Outcome of the Study	Critical Assessment (Pros & Cons)	Ref
Lactoferrin-tagged QD-based nanocapsules loaded with celecoxib and honokiol	Enhanced cytotoxicity against cancer cells and superior in vivo antitumor efficacy	Pros: effective delivery and enhanced drug action. Cons: complexity in formulation may impact manufacturing scalability.	[100]
Hybrid peptide conjugated with QDs and magnetic nanoparticles for HER2/neu-positive breast cancer	Enabled the detection and destruction of HER2/neu-positive breast cancer cells	Pros: dual function for detection and treatment. Cons: potential for off-target effects needs careful evaluation.	[101]
Anti-EGFR nanobody-conjugated InP/ZnS QDs loaded with aminoflavone	Enhanced tumor targeting and treatment efficacy in a xenograft model	Pros: targeted therapy shows high efficacy. Cons: nanobody use may face regulatory and production challenges.	[102]
Core/shell nanoprobes based on Fe₃O₄ MNPs and QDs targeting HER2	Enabled the in vivo multimodal imaging of tumors and the specific targeting of cancer cells	Pros: combines imaging and targeting, enhancing diagnostic precision. Cons: potential issues with nanoparticle aggregation and biocompatibility.	[103]
Quantum dots for the quantitative immunofluorescence of EGFR in breast cancer tissue arrays	High correlation with conventional immunohistochemistry, identifying the prognostic value of EGFR	Pros: provides quantitative data correlating well with established techniques. Cons: dependency on specific imaging equipment limits wider use.	[104]
Quantum dot-based immunofluorescent imaging for TOP2A in triple-negative breast cancer	Demonstrated TOP2A as an independent prognostic indicator in TNBC	Pros: high correlation in detection methods enhances diagnostic confidence. Cons: specialized imaging requirements may limit accessibility.	[105]
Quantum dots for the in situ molecular imaging of EGFR and collagen IV in TNBC	Identified negative correlation between EGFR and collagen IV, with prognostic value	Pros: enables detailed tissue analysis at the molecular level. Cons: the complex interpretation of dual molecular interactions.	[106]
Quantum dot-based double-color fluorescent imaging for Ki67 and HER2 co-expressions in breast cancer	Quantitatively assessed co-expressions and their impacts on prognosis	Pros: Allows detailed analysis of multiple markers simultaneously. Cons: Complexity in quantifying multiple signals accurately.	[107]
Quantum dot-based multiple imaging for Ki67 and cytokeratin in breast cancer	Developed method provided clear signal separation, quantification, and prognostic value	Pros: Enhances clarity and quantitative analysis in tissue samples. Cons: May require advanced imaging setups not available in all labs.	[108]
Quantum dot–antibody nanoprobes for monitoring notch pathway inhibition in breast cancer stem cells by capsaicin	Demonstrated effective targeting and apoptosis induction in breast cancer stem cells	Pros: the specific targeting of cancer stem cells could improve treatment outcomes. Cons: the complexity and specificity of pathway targeting may limit general applicability.	[109]
Quantum dots for imaging CCL5 and collagen IV in luminal B (HER2-) breast cancer	Demonstrated that the CCL5/collagen IV ratio has significant prognostic value for disease-free survival	Pros: provides valuable prognostic information for treatment planning. Cons: specialized applications may not translate to broader cancer types.	[110]
Niosomes with quantum dots and hyaluronic acid, loaded with paclitaxel and sodium oxamate for chemostarvation therapy.	Highly effective against breast cancer cells with minimal effects on normal cells; improved drug uptake and the induction of apoptosis in cancer cells.	Pros: dual drug delivery enhances therapeutic effects; specific targeting reduces collateral damage to normal cells. Cons: complexity in nanoparticle formulation; long-term in vivo efficacy and toxicity need extensive testing.	[111]

**Table 7:** Targeted theranostic with quantum dots in breast ancer.

QD Product and Its Properties	Outcome of the Study	Critical Assessment (Pros & Cons)	Ref
Quantum dots conjugated with monoclonal antibodies for HER2-receptor	Enabled the specific localization and bioimaging of HER2 receptors, leading to cancer cell apoptosis	Pros: specific targeting enhances therapy precision. Cons: potential for immunogenic responses or off-target effects.	[1]
Quantum dots conjugated with the peptide sequence LTVSPWY	Successfully targeted breast cancer cells in vitro and in vivo	Pros: peptide targeting offers a novel approach to cancer therapy. Cons: efficacy and safety in human trials remain to be established.	[2]
Quantum dots in lipid nanocarriers coupled with anti-EGF receptor aptamer	Delivered siRNAs and QDs effectively for tumor imaging and gene silencing	Pros: dual functionality for imaging and therapy. Cons: complexity in design may impact production and scalability.	[3]
CdSe/ZnS core/shell quantum dots conjugated with Herceptin	Specifically targeted and killed SK-BR3 breast cancer cells	Pros: high specificity for targeted cancer cell types. Cons: the stability and potential toxicity of cadmium content are concerns.	[9]
Dendrimer-functionalized carbon quantum dots from sweet lemon peel	Efficient gene delivery and selective cancer cell targeting in TNBC	Pros: biocompatible sourcing from natural materials. Cons: limited data on long-term effects and delivery efficiency.	[24]
Graphene quantum dots (GQDs) for photothermal and photodynamic therapy	Show promise in theranostic applications for breast cancer	Pros: versatile applications in therapy and imaging. Cons: long-term effects and biodistribution need further research.	[25]
Quantum dots conjugated with 1,25-dihydroxyvitamin D3 and MUC-1 antibodies	Predicted tissue-specific QD concentrations in mice for inflammatory breast cancer treatment	Pros: provides a basis for optimizing QD-based treatments. Cons: actual effectiveness in clinical settings to be verified.	[56]
Carbon quantum dots–quinic acid conjugate, nitrogen-doped	High tumor accumulation and excellent luminescent properties	Pros: strong luminescent properties suitable for imaging and therapy. Cons: more studies are needed to establish safety and efficacy in clinical applications.	[112]

**Table 8:** Quantum dots for metastasis prediction and inhibition in breast cancer.

QD Product and Its Properties	Outcome of the Study	Critical Assessment (Pros & Cons)	Ref
Quantum dot-DNA nanocomposites in electrochemical biosensor	High sensitivity for BRCA1 mutation detection in human serum, enhancing early breast cancer diagnostics	Pros: provides a sensitive and specific method for early genetic screening. Cons: requires further validation for routine clinical use.	[18]
Antibody-conjugated quantum dots with IGF1R antibody AVE-1642	The effective detection and downregulation of IGF1R, suggesting a traceable therapeutic approach	Pros: enhances both detection and therapeutic targeting. Cons: potential issues with antibody specificity and immune responses.	[19]
AMD3100-Ag2S quantum dot probe targeting CXCR4–CXCL12 axis	Predicted and inhibited metastasis effectively, with a photothermal effect reducing primary tumor size	Pros: combines diagnostic, therapeutic, and photothermal capabilities. Cons: complex synthesis and potential systemic toxicity need careful assessment.	[28]

**Table 9:** Cytotoxicity and therapeutic potential of quantum dots in breast cancer.

QD Product and Its Properties	Outcome of the Study	Critical Assessment (Pros & Cons)	Ref
Cadmium telluride quantum dots (QDs)	Induced epigenetic changes and activated stress response pathways in cancer cells	Pros: shows potential for inducing targeted cellular changes in cancer therapy. Cons: cadmium content raises significant toxicity and environmental concerns.	[10]
CdTe and CdTe/CdS core/shell QDs	Induced apoptosis in breast cancer cells in a dose-dependent manner	Pros: effective in inducing programmed cell death in cancer cells. Cons: cadmium toxicity remains a major drawback for clinical use.	[11]
Carbon quantum dots synthesized from bibenzoimidazolyl derivative	Exhibited significant antitumor activity against breast cancer cells with low toxicity to normal cells	Pros: high efficacy with lower toxicity to normal cells enhances therapeutic window. Cons: more data needed on long-term effects and biocompatibility.	[14]
CdS quantum dots synthesized using waste tea leaves	Induced cell death and arrested the cell cycle in breast cancer cells	Pros: the innovative use of waste materials in synthesis; shows effective cytotoxicity. Cons: potential environmental and health risks associated with cadmium.	[15]
Zinc oxide quantum dots (ZnO QDs), small-sized (8–10 nm)	Induced significant cytotoxicity and apoptosis in cancer cells at low concentrations	Pros: effective at low concentrations, indicating the potential for high therapeutic efficacy with minimal dosage. Cons: concerns about ZnO toxicity and accumulation in biological systems.	[114]
Graphene quantum dots (GQDs)	Induced apoptosis and cell cycle arrest specifically in estrogen receptor-positive breast cancer cells	Pros: specific action against estrogen receptor-positive cells could limit side effects. Cons: requires careful handling due to graphene’s properties and potential health impacts.	[115]

**Table 10:** Quantum dot applications in histochemistry and glycophenotype analysis.

QD Product and Its Properties	Outcome of the Study	Critical Assessment (Pros & Cons)	Ref
Quantum dots conjugated to folic acid (FA)	Effectively targeted and entered breast cancer cells, showing variation in internalization among cell types	Pros: targeted delivery to cancer cells via folate receptors. Cons: variable effectiveness across different cancer cell types may limit universal application.	[12]
CdTe quantum dots conjugated with lectins	Distinguished between normal and cancerous tissues based on glycoconjugate expression	Pros: provides a method for distinguishing tissue types without invasive methods. Cons: potential toxicity from cadmium-based materials.	[20]
Quantum dots conjugated with Cramoll lectin	Effectively labeled breast tissues, showing increased glucose/mannose residues in malignant tissues	Pros: useful for the detailed analysis of cancer tissue glycophenotypes. Cons: the specificity and sensitivity of lectin binding need further validation.	[21]
Alexa Fluor–quantum dot conjugates for multiplex immunoprofiling	Revealed the co-expression and spatial relationships of breast cancer biomarkers	Pros: enhances the understanding of biomarker relationships, aiding in therapy design. Cons: complexity in multiplex setup might limit routine use.	[22]
MPA-COOH-CdTe QDs with size-dependent cellular accumulation	ABC transporters modulate QDs toxicity, with smaller QDs inducing a greater transporter expression	Pros: insight into QD–cell interactions and transporter modulation. Cons: the inherent toxicity of CdTe could pose health risks.	[26]
Anti-mortalin antibody-conjugated quantum dots	Demonstrated the requirement of direct cell-to-cell contact for QD and mortalin transfer between cells	Pros: offers a novel method for studying protein transfers in cancer environments. Cons: limited by the need for direct contact, which may not be feasible in all therapeutic scenarios.	[57]

**Table 11:** Quantum dot-facilitated cell death monitoring and gene delivery in breast cancer.

QD Product and Its Properties	Outcome of the Study	Critical Assessment (Pros & Cons)	Ref
Copper sulfide quantum dots on nano-graphene oxide sheets	Enhanced cytotoxic effect against MCF-7 breast cancer cells with photothermal therapy	Pros: effective for targeted photothermal therapy. Cons: potential issues with nano-graphene oxide’s long-term biocompatibility and disposal.	[5]
Cancer cell membrane-coated biomimetic black phosphorus quantum dots	Inhibited tumor recurrence and metastasis, enhancing antitumor immune response	Pros: innovative approach to reprogramming the tumor microenvironment. Cons: the complexity and stability of biomimetic coatings need further study.	[6]
PEGylated graphene quantum dot nanoparticles conjugated with Herceptin and beta-cyclodextrin	Demonstrated efficient tumor targeting, controlled drug release, and enhanced antitumor activity	Pros: high efficiency in targeting and drug delivery. Cons: potential challenges in scaling up and ensuring consistent manufacturing.	[7]
Zinc(II) phthalocyanines–graphene quantum dot conjugates	Altered photophysical properties increased photodynamic therapy activity	Pros: improved the effectiveness of photodynamic therapy. Cons: photostability and interactions with biological tissues require further investigation.	[16]
Cationic morpholino-phthalocyanine conjugated to nitrogen and nitrogen–sulfur-doped graphene quantum dots	Improved ROS generation and enhanced therapeutic efficacy in combination therapy	Pros: enhanced the generation of reactive oxygen species for therapy. Cons: safety and specificity in clinical applications need validation.	[17]
Quantum dot–antibody conjugates for TIRF microscopy	The effective determination of drug concentrations against breast cancer stem cells with high-content imaging	Pros: high specificity and precision in imaging and analysis. Cons: technique may be limited to specialized research settings due to equipment requirements.	[23]
Graphene oxide quantum dot–polyethyleneimine complexes for gene delivery	Successful gene delivery and induced apoptosis in breast cancer cells	Pros: potential for targeted gene therapy applications. Cons: complexities involved in ensuring safe and efficient delivery across different cell types.	[27]
